# Gastroprotective effect of rhodanine and 2,4-thiazolidinediones scaffolds in rat stomachs by contribution of anti-apoptotic (BCL-2) and tumor suppressor (P53) proteins

**DOI:** 10.1038/s41598-024-51446-4

**Published:** 2024-01-19

**Authors:** Rozh Q. Ameen, Zahra A. Amin, Hiwa O. Ahmad, Diler D. Ghafur, Melodya G. Toma, Nyan Sabah, Muhammad Fakhir, Gardoon Abdulla

**Affiliations:** 1 Department of Pharmacy, Paitaxt Technical Institute, Erbil, Kurdistan Region Iraq; 2 Department of Clinical Analysis, College of Pharmacy, Hawler Medical University, Erbil, Kurdistan Region 44001 Iraq; 3 Department of Pharmaceutical Chemistry, College of Pharmacy, Hawler Medical University, Erbil, Kurdistan Region 44001 Iraq; 4 Department of Chemistry, College of Education, Salahaddin University, Erbil, Kurdistan Region Iraq; 5 Pharmacy Department, College of Medicine, University of Kurdistan- Hewlêr, Erbil, Kurdistan Region Iraq

**Keywords:** Chemical biology, Chemistry

## Abstract

In recent times, the methods used to evaluate gastric ulcer healing worldwide have been based on visual examinations and estimating ulcer dimensions in experimental animals. In this study, the protective effect of rhodanine and 2,4-thiazolidinediones scaffolds compared to esomeprazole was investigated in an ethanol model of stomach ulcers in rats. Pretreatment with experimental treatments or esomeprazole prevented the development of ethanol-induced gastric ulcers. The severity of the lesions and injuries was significantly lower than that of vehicle (10% Tween 80) treated rats. Significant and excellent results were obtained with the compound **6** group, with inhibition percentage and ulcer area values of 97.8% and 12.8 ± 1.1 mm^2^, respectively. Synthesized compounds **2**, **7** and **8** exhibited inhibition percentages and ulcer areas of 94.3% and 31.2 ± 1.1 mm^2^, 91. 3% and 48.1 ± 0. 8 mm^2^, 89. 5% and 57. 6 ± 1. 2 mm^2^, and 89. 1% and 60.3 ± 0. 8 mm^2^, respectively. These biological outcomes are consistent with the docking studies in which Compounds **7** and **8** showed remarkable binding site affinities toward human H+/K+-ATPase α protein (ID: P20648), rat H+/K+-ATPase α protein (ID: P09626), and Na+/K+-ATPase crystal structure (PDB ID:2ZXE) with binding site energies of − 10.7, − 9.0, and − 10.4 (kcal/mol) and − 8.7, − 8.5, and − 8.0 (kcal/mol), respectively. These results indicate that these test samples were as effective as esomeprazole. Likewise, immunohistochemical staining of antiapoptotic (BCL2) and tumor suppressor (P53) proteins showed strong positive marks in the10% Tween 80- treated group, opposing the mild staining results for the esomeprazole-treated group. Similarly, the staining intensity of the group treated with Compounds **2**–**8** was variable for both proteins.

## Introduction

Due to pepsin or gastric acid secretion, peptic ulcer disease is characterized by discontinuity in the GI tract's inner lining. The disease penetrates the muscularis propria layer of the stomach epithelium^1^ and typically affects the stomach and proximal duodenum; less frequently, the lower esophagus, distal duodenum, or jejunum are affected due to hypersecretory conditions, hiatal hernias, or ectopic gastric mucosa^2^. Most available gastroprotective medications work by inhibiting the harmful factors that neutralize acid secretion, such as antacids; H_2_ receptor blockers, such as ranitidine; anticholinergics, such as pirenzepine; proton pump inhibitors, such as omeprazole; and lansoprazole. However, using these antisecretory medications may result in negative side effects and ulcer recurrence^3^. Apoptosis was documented to have a role in the development of gastric mucosal ulcers, and the manifestation of p53 and Bcl-2 proteins was related to the proliferation of gastric cancer. Bcl-2 influences early gastric cancer, while p53 plays a role in middle- and late-stage gastric cancer^4^. Ethanol-induced gastric ulcers enhance the accumulation of oxygen-derived free radicals, which inter the apoptosis pathway, and the Bcl-2 protein attacks the release of pro-apoptotic factors; thus, their levels might be remarkably upregulated during gastric ulcer damage^5^. As preferred scaffolding for drug discovery, thiozothiazolidine-2–4-ones and rhodanine derivatives have been the focus of intensive research by organic chemists and biologists over the past two decades. Recent research revealed that these compounds exhibit a wide spectrum of medicinal characteristics^6^. The range of their biological action includes antifungal, anticancer, antidiabetic, anti-inflammatory, antiviral, anticonvulsant, and enzyme-inhibiting activities^7^. According to studies, the substitution of hydrophobic and aromatic groups at position 5 of thiozothiazolidine-2–4-ones and rhodanine increases biological activity^8^.

Due to the lack of research studies evaluating the biological characteristics of 5-substituted rhodanine and 2,4-thiazolidinedione derivatives, we decided to fill a knowledge gap and utilize the effect of 5-substituted rhodanine and 2,4-thiazolidinedione scaffolds on the treatment of gastric ulcers. Therefore, we synthetized derivatives of the moiety attached via a hydrocarbon aromatic linker at position C-5 of rhodanine and 2,4-thiazolidinediones by two different methods and tested their gastroprotective effect on ethanol-induced stomach ulcers in experimental rats by examining the stomach content, macroscopic stomach appearance and histology and immunohistochemistry of stomach tissues.

## Materials and methods

### Experimental section

All starting compounds were obtained from Fisher Scientific, Sigma-Aldrich, Acemec Biochemical, CHEM-LAB and Scharlau, used without any further purification. ^1^H-NMR and ^13^C-NMR spectra were recorded on 400 MHZ spectrometer and FT-IR instrument were used for identification.

### Chemistry

#### General procedure for synthesis of compounds

**Figure 1:**
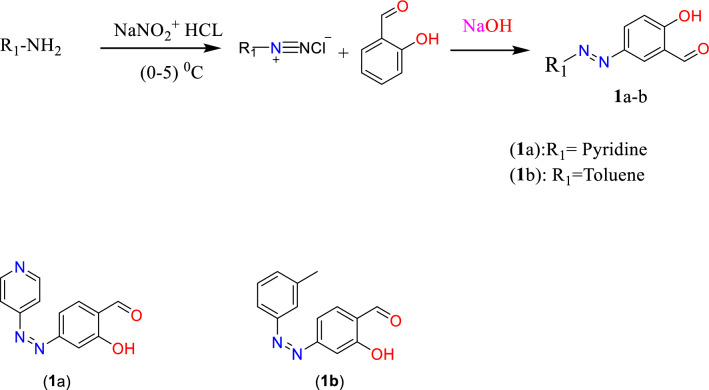
Stepwise chemical reaction for synthesis of substituted Aldehydes **1**a–b.

Commercially available aromatic amine compounds were dissolved in HCl (0 °C) and 7% NaNO_2_ (solution A) mixed with 2-hydroxybenzaldehyde and 4% NaOH (solution B) at 0–5 °C^9^. Based on the demonstrated results, the product was collected by filtration, washed with water, and recrystallized from the appropriate solvents (25 ml) to generate Compounds 1a and 1b. (Fig. 1).

**Figure 2:**
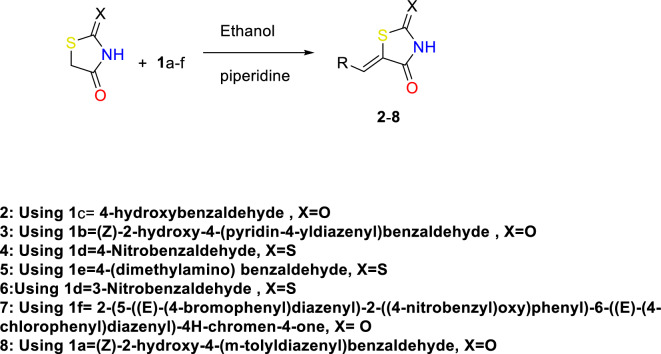
Knoevenagel condensation reaction of aromatic aldehydes and ketone with thiazolidine-2,4-dione and 2-thioxothiazolidin-4-one.

On the other hand, commercially available 2-thioxothiazolidin-4-one and rhodanine were reacted with substituted aldehydes **1**a–f in ethanol in the presence of piperidine in a round bottom flask equipped with a magnetic stirrer and reflux condenser. The mixture was stirred at 150 °C. The solid products were filtered off and washed with ethanol. The pure compounds were collected and dried. The product was recrystallized by ethanol. The structures of isolated products **2**–**8** were confirmed by spectral data (Fig. 2)^6,10^.

### Induction of acute gastric lesion by ethanol

The gastroprotective part of the experiment was performed at the animal house laboratory, College of Pharmacy, Hawler Medical University. The whole animal experiments were performed according to the National Institutes of Health's Guide for Care and Use of Laboratory Animals and the Animal Research: Reporting in Vivo Experiments (ARRIVE) Guidelines and under permission of ethically approved protocols of the College of Pharmacy’s ethics Committee, Hawler Medical University (Ethics number: HMU.PH. EC, 190720-110). All methods are reported in accordance with ARRIVE guidelines (https://arriveguidelines.org). The animal procedure was performed following a method by Mahmood et al.^11^ with minor changes. Fifty-five male rats weighing 200–280 g were fasted 48 h prior to the experiment, but drinking water was permitted until two hours before the experiment. Animals were divided into the following groups: Group 1 received 10% Tween 80, Group 2 received 20 mg/kg esomeprazole, and Groups 3–9 received rhodanine and 2,4-thiazolidinedione derivatives named Compounds **2–8**, respectively. Then, absolute ethanol was used as an ulcer inducer (5 ml/kg), and animals were left for one more hour and then sacrificed with an intraperitoneal injection of ketamine (80 mg/kg) + xylazine (20 mg/kg) into the lower quadrant of the animal's abdomen. Stomachs were collected and observed macroscopically for the appearance of gastric ulcers, stored in 10% formalin and examined for histopathology and immunohistochemistry. Stomach content was collected, centrifuged and then used for measurement of pH and mucus weight.

### In silico molecular docking studies

**Figure 3:**
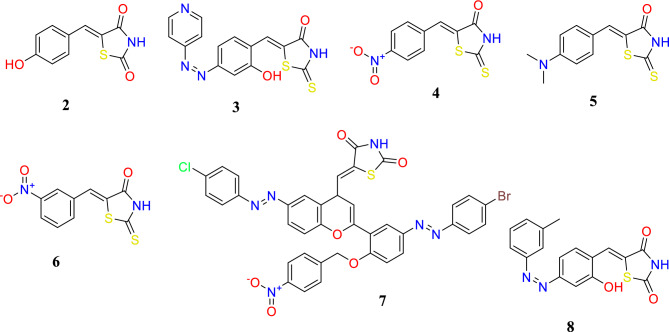
Two-dimensional (2-D) structures of the ligand molecules (**2**–**8**).

The two-dimensional (2-D) structures of the ligand molecules (**2**–**8**) (Fig. 3) were built using ChemDraw professional 16.0, converted to three-dimensional (3-D) structures using the Chem3D 16.0 module and saved as PDB format structures (http://www.cambridgesoft.com/).

The ligand was optimized by adding hydrogens, and the pdbqt format of the ligands was prepared with AutoDock Tools 1.5.7^12^. The ligand molecules were then used as input for AutoDock Vina (https://vina.scripps.edu/) to carry out the docking simulation.

Protein molecules of the human gastric H+/K+-ATPase α chain (Swiss-Prot ID: P20648), rat H+/K+-ATPase α protein (Swiss-Prot ID: P09626), and Na+/K+-ATPase crystal structure (PDB ID:2ZXE) were retrieved from the protein data bank (http://www.rcsb.org/pdb/)^13^. The grid dimensions were set at 3.095 × 10.4 × 6.75 (Swiss ID: P20648), 4.52 × 5.30 × 2.63 (Swiss ID: P09626), and 147.45 × 17.85 × − 4.48 (PDB ID: 2ZXE) according to the coordinates x, y, and z, respectively, determined by BIOVIA Discovery Studio 2021^14^, for the target binding sites. The water molecules were removed from the receptors, and polar hydrogen and Kollman charges were added. The pdbqt format of the receptors was prepared by AutoDock Tools 1.5.7. AutoDock Vina was compiled and run under Windows 10.0. Professional operating system. Discovery Studio 2021 was used to deduce the pictorial representation of the interaction between the ligands and the target protein. Traditional calculations can be performed using the basic equations for enzymatic kinetics from a Lineweaver–Burk assay, which is extrapolated on 2D to determine the enzyme-inhibitor complex’s inhibition constant (Ki). Sophisticated arithmetic and analytical in silico algorithms have been proposed to compute the inhibition constant (*K*i) parameter^15^.

#### ADME prediction

Prediction of pharmacokinetics and physicochemical parameters plays a key role in drug design^16^. The evaluation of drug-likeness properties was evaluated for Compounds **2**–**8** using SwissADME (http://www.swissadme.ch/), (https://biosig.lab.uq.edu.au/pkcsm/prediction) and admetSAR (http://lmmd.ecust.edu.cn/admetsar2)^17^. Drug-like molecules must obey Lipinski’s rule of five (RO5) as follows: the molecular weight MW of the active oral drug should be ≤ 500 Da; the log P should be < 5; the number of hydrogen bond acceptors should be nOH ≤ 10; the number of hydrogen bond donors nOHNH should be ≤ 5; and the number of rotatable bonds should be ≤ 10^18^.

### Ethical approval

Animal procedures were performed under the National Institutes of Health's Guide for Care and Use of Laboratory Animals and the Animal Research: Reporting in Vivo Experiments (ARRIVE) Guidelines and under permission of ethically approved protocols of College of Pharmacy’s ethics Committee, Hawler Medical University (Ethics number: HMU.PH.EC, 190720-110).

## Results and discussion

### Compound identification

#### Synthesis of (*Z*)-5-(4-hydroxybenzylidene)thiazolidine-2,4-dione (2)


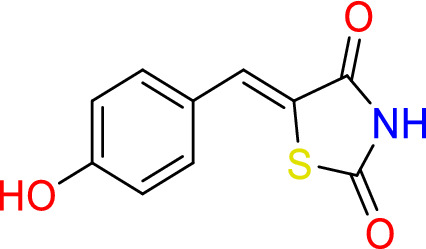


Thiazolidine-2,4-dione (0.5 g, 4.3 mmol), piperidine (0.3 ml, 0.3 mmol) and 4-hydroxybenzaldehyde (0.5 g, 4.1 mmol) were added to 20 ml of ethanol, reflex for 24 h. at 150 °C.

Yield = 80.0%, m.p: 205–207 °C, ^1^H– NMR (400 MHz, d6-DMSO): δ 12.7 (s, 1H, N**H**), 7.8 (s, 1H, CHCS), 7.7 (dd, J = 8.7, 5.5 Hz, 2H, C**H**CC**H**), 7.4 (t, J = 8.8 Hz, 1H, C**H**C).

13C NMR (101 MHz, d6-DMSO): δ 167.8 (**C**=O), 167.4 (**C**=O), 164.5 (**C**–OH), 161.5 (H**C**=C), 132.5 (Ar), 132.4 (Ar), 130.6 (Ar), 123.4 (Ar), 116.6 (Ar), 116.4 (S). IR (neat): vmax = 1727 cm^−1^ (C=O), 1689 cm^−1^ (C=O), 3031 cm^−1^ (NH), 3112 cm^−1^ (OH), 1581 cm^−1^ (C=C).

#### Synthesize of 5-((Z)-2-hydroxy-4-((z)-pyridin-4-yldiazenyl)benzylidene)-2-thioxothiazolidin-4-one (3)


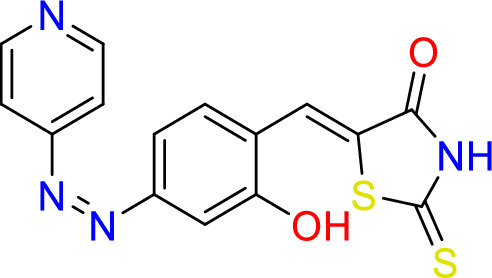


2-thioxothiazolidin-4-one (0.5 g, 3.8 mmol), and (*Z*)-2-hydroxy-4-(pyridin-4-yldiazenyl) benzaldehyde (0.5 g, 2.2 mmol) (**1a**) were added to 20 ml of ethanol, reflex for 24 h at 150 °C.

Yield = 83.0%, Mp: 267–269 °C. ^1^H-NMR (400 MHz, d6-DMSO): δ 13.13 (s, 1H, N**H**), δ 10.40 (s, 1H, O**H**), δ 8.14 (d, J = 4.4 Hz, 2H, C**H**NC**H**), δ 7.50 (s, 1H, C**H**C), 7.44 (d, J = 8.6 Hz, 2H, C**H**CNC**H**), 7.10 (s, 1H, C**H**CS), 6.92 (d, J = 8.6 Hz, 2H, C**H**C**H**C). 13C– NMR (101 MHz, d6- DMSO): δ 196.2 (CS), 170.4 (CO), 160.8 (**C**N=N**C**), 152.7 (2 × N**CC**OH), 140 (**C**HCS), 133.5 (2X **C**HN**C**H), 133.1 (**C**HCOH), 132.7 (**C**HCCH), 124.5 (CHC**C**H), 121.7 (**C**N), 117.0 (2 × **C**HCN**C**H). IR (neat): vmax = 2935 cm^−1^ (NH), 3027 cm^−1^ (OH), 1739 cm^−1^ (CN), 1670 cm^−1^ (C=O), 1550 cm^−1^ (C=C).

#### Synthesize of (*Z*)-5-(4-nitrobenzylidene)-2-thioxothiazolidin-4-one (4)


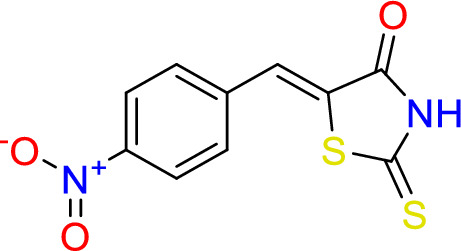


2-thioxothiazolidin-4-one (0.5 g, 3.8 mmol), piperidine (0.3 ml, 0.3 mmol), and 4-Nitrobenzaldehyde (0.6 g, 3.9 mmol) were added to 20 ml of water, reflex for 24 h. at 150 °C.

Yield = 76.0, M.P. = 260–261 °C. ^1^H -NMR (400 MHz, d_6_-DMSO): δ 14.04 (s, 1H, N**H**), 8.35 (d, J = 8.8, 2H, **H**CCC**H**), 7.87 (d, J = 8.8, 2H, **H**CCC**H**), 7.75 (s, 1H, C**H**C). ^13^C– NMR (101 MHz, d6- DMSO): δ 195.3 (CS), 169.3 (CO), 147.5 (**C**NO_2_), 139.2 (CHCS), 131.3 (2xC), 138.5 (C),129.9 (CS), 124.3 (2xC). IR (neat): *v*max = 1720 cm^−1^ (C=O), 3027 cm^−1^ (NH), 1589 cm^−1^ (C=C).

#### Synthesis of (Z)-5-(4-(dimethylamino)benzylidene)-3-thioxoisothiazolidin-4-one (5)


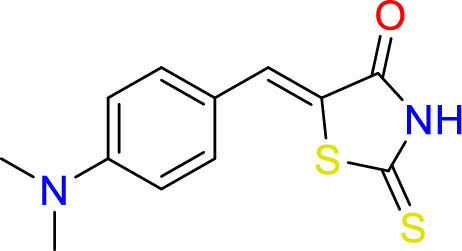


2-thioxothiazolidin-4-one (1.0 g, 9.5 mmol), piperidine (0.3 ml, 0.3 mmol), and 4-(dimethylamino) benzaldehyde (1.3 gm, 8.7 mmol) were added to 20 ml of ethanol in a round bottom flask, reflex for 3 h. at 150 °C.

Yield = 87%, Mp: 295–98 °C,^1^H- NMR (400 MHz, d_6_-DMSO): δ 13.54 (s, 1H, N**H**), 7.50 (s, 1H, **C**H), 7.40 (d, *J* = 8.7 Hz, 2H, Ar), 6.80 (d, *J* = 8.7 Hz, 2H, Ar), 3.02 (s, 6H, C**H**_3_). ^13^C -NMR (126 MHz, d_6_-DMSO): δ 195.5 (**C**=S), 170.4 (**C**=O), 151.6 (**C**NCH_3_), 133.3 (**C**HCS), 120.1 (Ar), 117.6 (Ar), 111.9 (C–S) 43.4 (2 × **C**H_3_). IR (neat): vmax = 3150 cm^−1^ (NH), 1677 cm^−1^ (C=O), 1561 cm^−1^ (C=S), 1519 cm^−1^ (C=C), 1250 cm^−1^ (C–N).

#### Synthesize of (*Z*)-5-(3-nitrobenzylidene)-2-thioxothiazolidin-4-one (6)


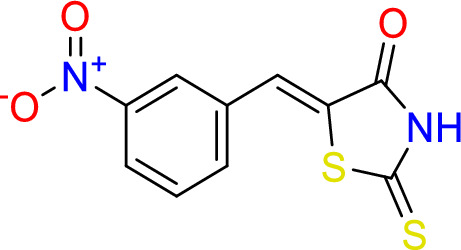


2-thioxothiazolidin-4-one (0.5 g, 3.8 mmol), piperidine (0.3 ml, 0.3 mmol) and 3-Nitrobenzaldehyde (0.5 ml, 3.3 mmol) were added to 20 ml of water, reflex for 24 h at 150 °C.

Yield = 83%, Mp: 259–261 °C, ^1^H -NMR (400 MHz, d6-DMSO): δ 14.03 (s, 1H, N**H**), 8.47 (s, 1H, CC**H**CNO_2_), 8.32 (dd, J = 8.2, 1.5 Hz, 1H, C**H**C), 8.02 (d, J = 7.8 Hz, 1H, C**H**CNO_2_CH), 7.84 (m, 2H, **H**C=C, overlapped C**H**CNO_2_CH). ^13^C– NMR (101 MHz, d6- DMSO): δ 195.2 (C=S), 169.3 (CO), 148.3 (**C**NO_2_), 135.7 (**C**HCS), 134.6 (Ar), 131.0 (Ar), 128.9 (Ar), 128.6 (Ar), 124.8 (Ar), 124.6 (C–S). vmax = 3235 cm^−1^ (NH), 1689 cm^−1^ (C=O), 1596 cm^−1^ (C=S), 1523 cm^−1^ (C=C), 1222 cm^−1^ (C–N).

#### Synthesis of (*Z*)-5-((2-(5-((*E*)-(4-bromophenyl)diazenyl)-2-((4-nitrobenzyl)oxy)phenyl)-6-((E)-(4-chlorophenyl)diazenyl)-4H-chromen-4-yl)methylene)thiazolidine-2,4-dione (7)


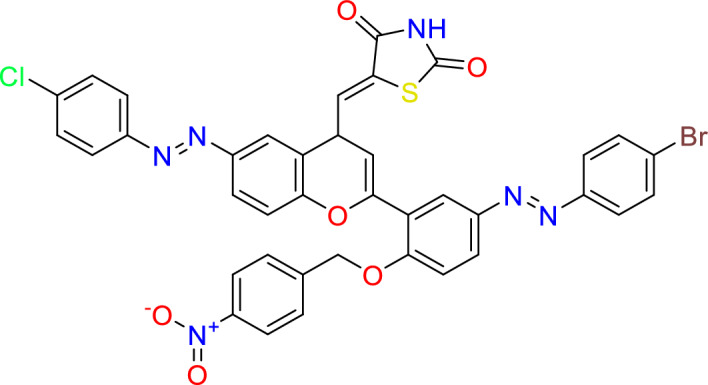


Thiazolidine-2,4-dione (1.0 g, 8.5 mmol), piperidine (0.3 ml, 0.3 mmol), and 2-(5-((E)-(4-bromophenyl)diazenyl)-2-((4-nitrobenzyl)oxy)phenyl)-6-((E)-(4-chlorophenyl)diazenyl)-4H-chromen-4-one (1.0 g, 1.2 mmol) (obtained from Mzgin and Farouq)^19^ were added to 50 ml of ethanol in a round bottom flask, reflex for 4 h. at 150 °C.

Yield = 65%, Mp: 220–222 °C 1H- NMR (400 MHz, DMSO): δ 13.73 (s, 1H, N**H**), 8.14 (d, J = 2.3 Hz, 1H, C**H**), 8.12 (d, J = 2.3 Hz, 1H, C**H**), 8.05 (d, J = 2.2 Hz, 1H, C**H**), 8.03 (s, 1H, C**H**), 7.81 (s, 1H, vinylic C**H**), 7.74 (td, J = 8.3, 1.3 Hz, 4H, C**H**), 7.64–7.47 (m, 6H, C**H**), 7.34–7.24 (m, 2H, C**H**), 6.85 (s, 1H, C**H**), 6.76–6.69 (m, 1H, C**H**), 5.41 (s, 2H, C**H**_2_O).

13C NMR (101 MHz, DMSO): δ 167.7 (C=O), 167.3 (C=O), 159.7 (C–O), 156.7 (C–O), 147.5 (C–O), 146.0 (2xCN), 145.8(CN), 135.0 (**C**CH_2_), 134.0 (H**C**=C), 132.9 (CCl), 132.6 (2xCH), 130.8 (C), 130.4 (2 × CH), 129.9 (CH), 129.8 (3xCH), 128.7 (2xCH), 128.7 (CH), 128.6 (CH), 128.1 (C), 126.4 (C), 125.5 (CH), 124.7 (C), 123.7 (CH), 122.9 (CH), 121.8 (CH), 120.8 (CH), 119.3 (C), 119.2 (C), 117.4 (CH), 113.9 (**C**S), 113.6 (**C**CH), 69.8 (**C**H_2_). vmax = 3031 cm^−1^ (NH), 1751 cm^−1^ (C=O), 1704 cm^−1^ (C=O), 1592 cm^−1^ C=C).

#### Synthesize of 5-((Z)-2-hydroxy-4-((Z)-m-tolyldiazenyl)benzylidene)thiazolidine-2,4-dione (8)


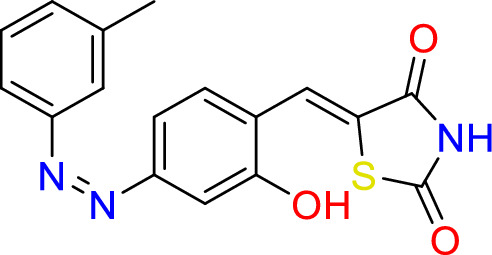


Thiazolidine-2,4-dione (2.0 g, 17.0 mmol), piperidine (0.3 ml, 0.3 mmol) and (Z)-2-hydroxy-4-(m-tolyldiazenyl)benzaldehyde (2.0 g, 8.3 mmol) were added to 20 ml of ethanol, reflex for 24 h at 150 °C.

Yield = 77%, Mp: 260–262 °C. ^1^H -NMR (400 MHz, d6-DMSO): δ 10.25 (s, 1H, OH), δ 7.71 (d, J = 8.6 Hz, 2H, C**H**CNC**H**), δ 7.59 ((s, 1H, vinylic H), δ 7.50- 7.53 (m, 3H, C**H**CC**H and C**H C**H** CH), δ 7.43 (d, J = 8.6 Hz, 2H, C**H**CCH3), δ 6.90 (d, J = 8.6 Hz, 2H, C**H**COH). 13C– NMR (101 MHz, d6- DMSO): δ 171.2 (CO), 168.7 (CO), 159.30 (**C**OH), 140.32 (H**C**=C),131.9 (**C**N=**C**N), 130.52 (**C**H**CC**H),129.90 (**C**CH), 128.6 (**C**H), 127.8 (**C**H), 124.5 (CH), 119.0 (2 × **C**H),116.2 (2x**C**H), 24.1 (**C**H_3_). IR (neat): vmax = 2938 cm^−1^ (NH), 3401 cm^−1^ (OH), 1739 cm^−1^ (C=O), 1670 cm^−1^ (C=O), 1550 cm^−1^ (C=C).

Rhodanine and 2,4-thiazolidinediones scaffolds are very important in drug design and discovery due to their ease of synthesis and numerous biological activities^20^. Commercially available rhodanine and 2,4-thiazolidinedione were mixed with substituted aldehyde and ketone under Knoevenagel condensation conditions. The chemical structures of the products **2**–**8** were established on the basis of IR, ^1^H-NMR, and ^13^C-NMR spectral data.

The IR spectra of N–H for thiazolidine-2,4-dione and 2-thioxothiazolidin-4-one derivatives **2**–**8** showed absorption bands in the range of 2935–3235 cm^−1^. The band in a range 1519–1592 cm^−1^ assigned to C=C functions. The characteristic absorption band at 1727, 1689 cm^−1^ attributed for Compound **2**, at 1751, 1704 for Compound **7**, and at 1739, 1670 cm^−1^ for Compound **8** due to (2C=O). In the same manner, 2-thioxothiazolidin-4-one derivatives **3**, **4**, **5**, and **6** showed characteristic absorption bands at 1670, 1720, 1677, 1689 cm^−1^, respectively which belong to a single C=O.

In ^1^H-NMR spectra, all the products 2–7 except Compound 8 showed characteristic singlet signals in the region 12.7–14.04 ppm, referred to as the –NH protons. The singlet signal of –NH of Compound 8 disappeared due of the Deuterium exchangeable. One singlet signal demonstrated approximately 7.5–7.84 ppm for the benzylidene proton of vinyl carbon (CH=C) that supported the occurrence of Knoevenagel reaction between thiazolidine-2,4-dione and 2-thioxothiazolidin-4-one 2–8 and the selected aromatic aldehydes. Concerning ^13^C NMR of the **thiazolidine-2,4-dione** 2, 7, and 8, the appearance of two signals at 167–171 ppm confirmed the presence of olefinic carbons. Considering Compounds 3–6, the 13C NMR spectra exhibited separate peaks for C=S and C=O ranging 195–196 and 169–17 ppm, respectively.

### In vivo animal model

To assess the mechanisms of action of newly synthesized compounds, animal models are usually used with standards analogous to clinical human trials. In this study, the effect of Rhodanine and 2,4-thiazolidinediones scaffolds derivatives were examined to evaluate their gastroprotective activity against ethanol-induced stomach injury. The results were compared to the Esomeprazole is a S-isomer of omeprazole; a medication treats peptic ulcer disease and reduces stomach acid and is as effective as proton pump inhibitors and works through H+/K+-ATPase in the parietal cells of the stomach^21^. Oral pretreatment with 20 mg/kg esomeprazole (ulcer negative group) two hours earlier than absolute ethanol was significantly effective against ulcer production, as it generates inhibition percentage and ulcer urea (77.3% and 124.1 ± 1.3 mm^2^, respectively) compared to the 10% Tween 80 (ulcer positive group) (550 ± 0.9 mm^2^); however, some of the experimental treatment groups showed more significant results in comparison to the negative control group and much better results than the esomeprazole group, as shown in Table 1. The most significant effects were shown by the Compound **6** group with inhibition percentage and ulcer area (97.8% and 12.8 ± 1.1 mm^2^, respectively). Compounds **2**, **7** and **8** had inhibition percentages and ulcer areas of 94.3% and 31.2 ± 1.1 mm^2^, 91.3% and 48.1 ± 0.8 mm^2^, 89.5% and 57.6 ± 1.2 mm^2^, and 89.1% and 60.3 ± 0.8 mm^2^, respectively. No significant changes were observed in the stomach mucus weight data, while the pH of the stomach was significantly changed from alkaline (8.5 ± 0.4 mEq/I) for the ulcer-positive group to acidic and strongly acidic in the esomeprazole and treatment groups, as shown in Table 1.

**Table 1:** Gastroprotective effect of test samples on stomach ulcer measurements.

Pretreatment		Ulcer area (mm2)	Inhibition (%)	Mucus weight (mg)	pH (mEq/l)
Vehicle	10% Tween 80	550.0 ± 0.9	…	0.4 ± 0.3	8.5 ± 0.4
Esomeprazole	20 mg/kg	124.1 ± 1.2*	77.3	1.5 ± 0.4	3.8 ± 0.5*
C2	50 mg/kg	48.1 ± 0.8*	91.3	1.3 ± 0.7	3.5 ± 0.3*
C3	50 mg/kg	444.4 ± 0.3	19.3	1.2 ± 1.0	2.3 ± 0.9
C4	50 mg/kg	194.4 ± 0.1*	64.7	1.2 ± 0.2	5.7 ± 0.4*
C5	50 mg/kg	156.6 ± 0.9*	71.6	0.7 ± 0.2	6.3 ± 0.9*
C6	50 mg/kg	12.8 ± 1.1*	97.8	1.1 ± 0.9	3.4 ± 0.5*
C7	50 mg/kg	57.6 ± 1.2*	89.5	1.1 ± 0.7	3.8 ± 0.6*
C8	50 mg/kg	60.3 ± 0.8*	89.1	1.3 ± 0.6	3.6 ± 0.7*

*C* Compound; (*) Indicates significance when compared with the 10% Tween 80 group at *p* ≤ 0.05.

**Figure 4:**
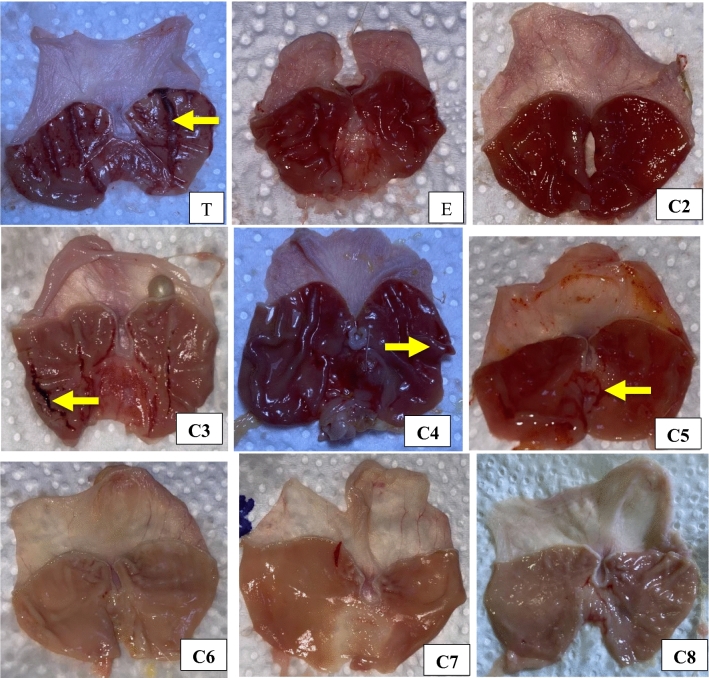
Macroscopic appearance of gastric mucosa of rats; (E) esomeprazole group shows mild injury compared to ulcer control group (T) which shows moderate to severe mucosal lesions. Similarly fewer severe lesions (yellow arrow) were shown by **3**, **4** and **5** treated rats even though mild lesion were seen in **7**. Oppositely, **2**, **6**, and **8** groups shows flattening of mucosal folds with almost no injuries.

The gross appearance of stomachs confirmed the previous data, in which rats treated with esomeprazole showed remarkably fewer gastric lesions than those in the 10% Tween group. Thus, proton pump inhibitors, involving esomeprazole, efficiently counteract gastric lesions and promote the healing of stomach and duodenal ulcers^22^, and previous research has shown that esomeprazole preserves gastric mucosa from NSAID-induced damage by a direct/indirect decrease in oxidative injury in tissues^23^. Correspondingly, rats administered Groups **2**–**8** showed variable results, especially Groups **2** and **6**, which showed less or no damage in the mucosa of surface epithelia; however, more gastric lesions at the submucosal lining were observed in Groups **3**, **4**, **5**, **7** and **8**, as shown in Fig. 4. These results agree with previous studies in which the in vivo gastroprotective activity of some rhodanine derivatives was recorded^24^, and other derivatives of these compounds were demonstrated to protect the intestine against injury and stress in experimental rats^25^. Similar significant antiulcer results were shown by pyrazoline and thiazolidinone derivatives in rat models of ethanol-induced ulcers and pylorus ligation methods^26^ Generally, thiazolidin-4-one derivatives have been well documented to exhibit many other biological activities, such as antidiabetic, anticancer, antioxidant, analgesic, anti-inflammatory, anticonvulsant and antimicrobial activities^27^. Additionally, safer effects were observed with thiazolidin-4-one derivatives than diclofenac, which causes liver, gastric, blood and renal toxicities^28^.

**Figure 5:**
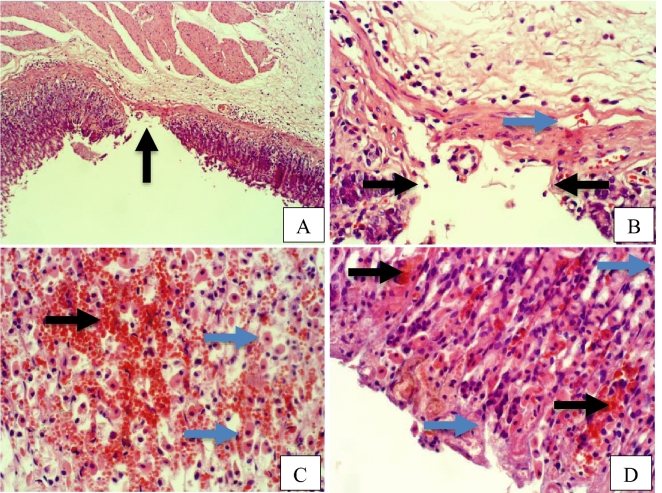
(**A**–**D**): Rat stomachs of ulcer positive group (10% Tween 80), (**A**) Showed classical mucosal ulcer (black arrow) H&E 100x. (**B**) Showed gastric mucosal ulcer (black arrow), and edema in muscularis mucosa layer (blue arrow). (**C**) Showed massive interstitial hemorrhages in gastric mucosa (black arrow), with hypertrophy of parietal cells (blue arrow). (**D**) Showed interstitial hemorrhages in gastric mucosa (black arrow), with vacuolar degeneration of chief cells (blue arrow) H&E 400x.

**Figure 6:**
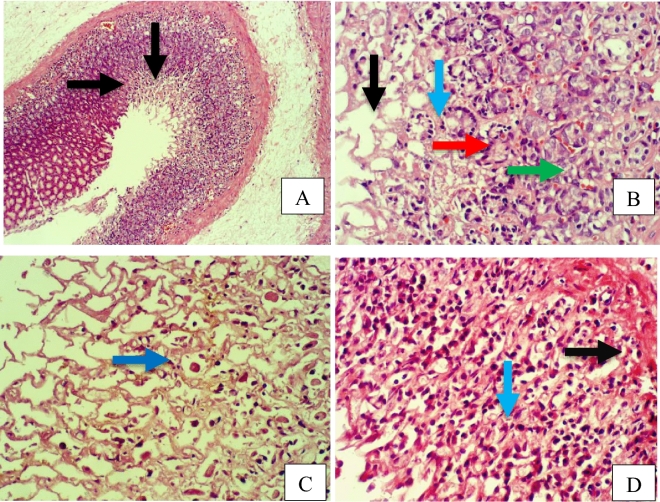
A&B: Stomach, compound 2 treated group, (**A**) Showed erosions at mucosa layer (black arrow). H&E 100x. (**B**) Showed erosions in gastric pit (black arrow), coagulative necrosis in gastric gland (blue arrow), infiltration of inflammatory cells (red arrow), vacuolar degeneration in chief cells (green arrow). H&E 400x. E&F: Stomach, compound 3 treated group, (**C**) Showed coagulative necrosis in the tips of gastric mucosa (black arrow), with slough and desquamation as cellular debris (blue arrow) H&E 400x. (**D**) Showed erosions in gastric mucosa (black arrow), coagulative necrosis in gastric gland (blue arrow), and vacuolar infiltration (red arrow) H&E 400x.

**Figure 7:**
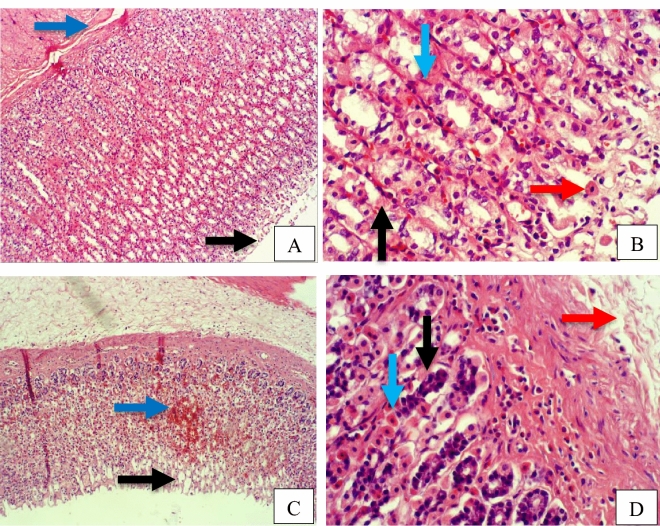
Stomach, Compound 4 treated group, (**A**) Showed erosions gastric pit (black arrow), edema in muscularis mucosa layer (blue arrow) H&E 100x. (**B**) Showed erosions in gastric mucosa (black arrow), coagulative necrosis in chief cells (blue arrow), and hypertrophy in parietal cells (red arrow) H&E 400x. C&D: Stomach, compound 5 group. (**C**) Showed erosions gastric pit (black arrow), hemorrhage between gastric glands (blue arrow) H&E 100x. (**D**) Showed mucinous degeneration in chief cells (black arrow), hypertrophy of parietal cells (blue arrow), edema in muscularis mucosa (red arrow).

**Figure 8:**
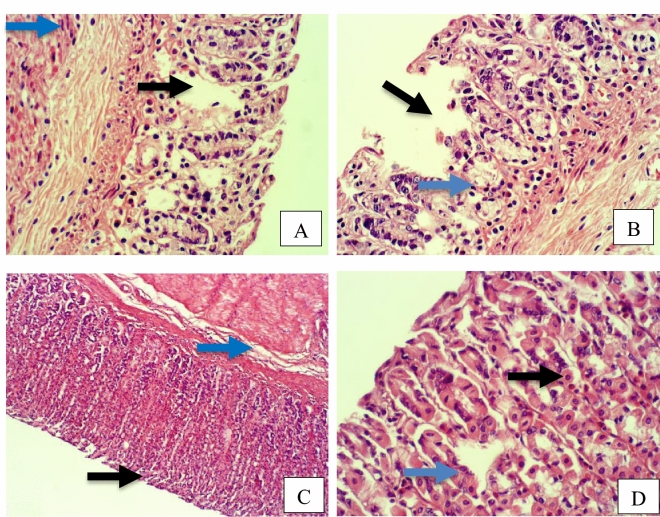
A&B: Stomach, compound 7 treated group. (**A**) Showed erosions in gastric mucosa with complete losing of mucosa layer in few places (black arrow), and fibrocytes hypertrophy (blue arrow) H&E 400x. (**B**) Showed erosions in gastric mucosa with complete losing of mucosa layer in few places (black arrow), and vacuolar degeneration of gastric gland cells (blue arrow). H&E 400x. C&D: Stomach, compound 8 treated group, (**C**) Showed normal gastric mucosa (black arrow), and edema in muscularis mucosa layer (blue arrow) H&E 100x. (**D**) Showed hypertrophy of parietal cells (black arrow), and necrosis in chief cells (blue arrow) H&E 400x.

**Figure 9:**
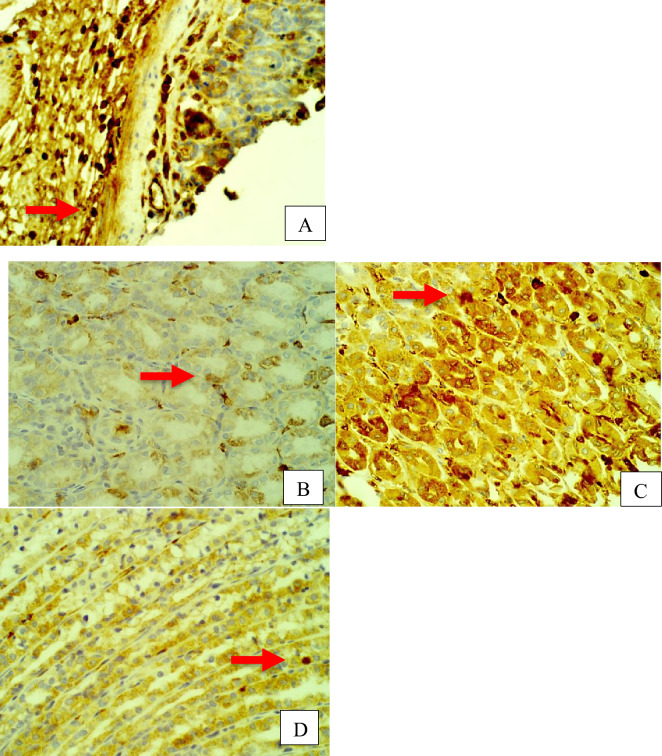
Stomach, IHC *BCL2* -ab 400x. (**A**) 10% Tween 80 treated group showed strong positive staining with *BCL2* antibodies in the cytoplasm of gastric mucosa at the edges of ulcer as golden-brown batches (red arrow). (**B**) compound 2 group showed few weakly positive staining with *BCL2* antibodies in the cytoplasm of parietal cells as golden-brown granules (red arrow). (**C**) compound 3 group showed strong positive staining with *BCL2* antibodies in the cytoplasm of parietal cells as golden-brown granules (red arrow). (**D**) compound 4 group showed positive staining with *BCL2* antibodies in the cytoplasm of chief cells as golden-brown granules (red arrow).

**Figure 10:**
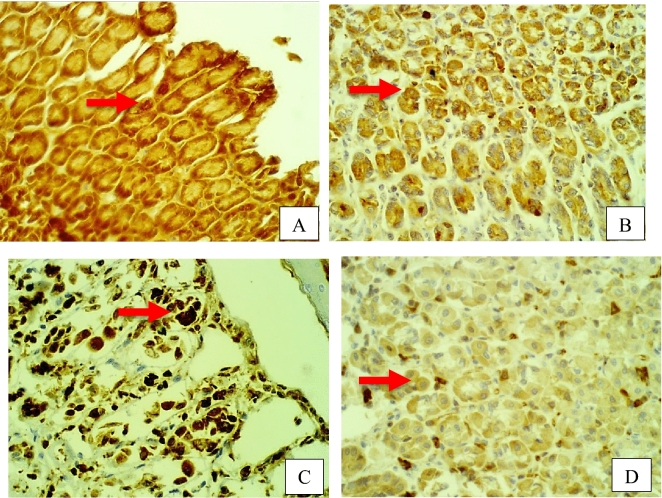
Stomach, IHC *BCL2* -ab 400x. (**A**) compound 5 group showed strong positive staining with *BCL2* antibodies in the cytoplasm of gastric gland as golden-brown granules (red arrow). (**B**) compound 6 group showed strong positive staining with *BCL2* antibodies in the cytoplasm of gastric gland as golden-brown granules (red arrow). (**C**) compound 7 group showed strong positive staining with *BCL2* antibodies in the cytoplasm of gastric gland as golden-brown granules (red arrow). (**D**) compound 8 group showed a few weak positive staining with *BCL2* antibodies in the cytoplasm of parietal cells as golden-brown granules (red arrow).

**Figure 11:**
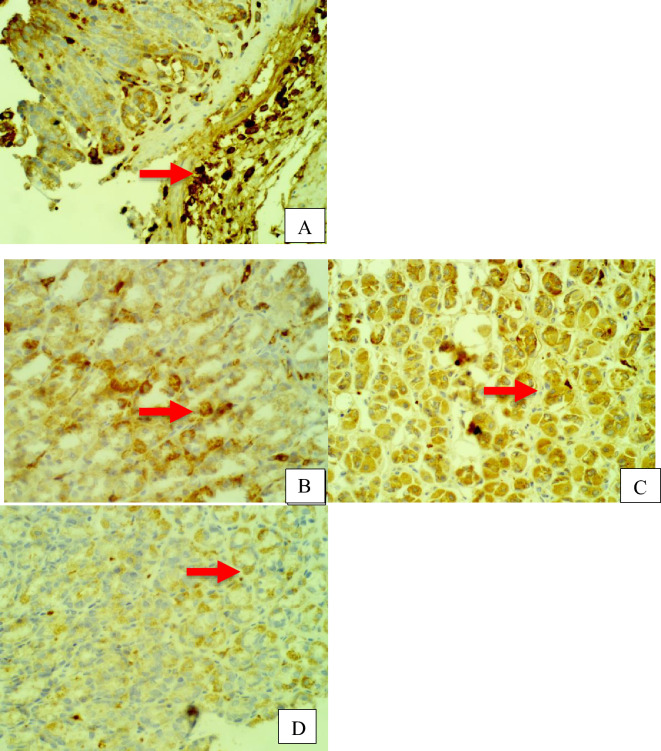
Stomach, IHC *P53* -ab. 400x. (**A**) 10% Tween 80 treated group showed strong positive staining to gastric mucosa at the edges of ulcer as cytoplasmic patches (red arrow) with *P53* antibodies. (**B**) Compound 2 group showed few weakly positive staining to cells of gastric gland (red arrow) with *P53* antibodies. (**C**) Compound 3 group showed few weakly positive staining to parietal cells of gastric gland (red arrow) with *P53* antibodies. (**D**) Compound 4 group showed few weakly positive staining to chief cells of gastric gland (red arrow) with *P53* antibodies.

**Figure 12:**
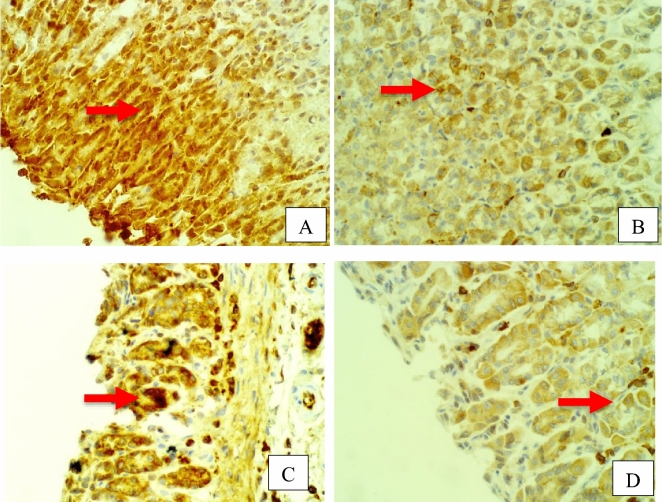
Stomach, IHC *P53* -ab. 400x. (**A**) Compound **5** group showed few weakly positive staining to cells of gastric gland (red arrow) with *P53* antibodies. (**B**) Compound 6 group showed few positive staining to cells of gastric gland (red arrow) with *P53* antibodies. (**C**) Compound 7 group showed strong positive staining to chief cells of gastric gland (red arrow) with *P53* antibodies. (**D**) Compound 8 group showed strong positive staining to parietal cells of gastric gland (red arrow) with *P53* antibodies.

Furthermore, rat stomach sections stained with hematoxylin and eosin were treated with 10% Tween 80 and showed intense edema with leucocyte infiltration. Severe interstitial hemorrhages in the mucosal layers with hypertrophy of parietal cells accompanied the degeneration of chief cells, as shown in Fig. 5. In contrast, esomeprazole-treated stomachs displayed normal histopathology, including layers of muscularis mucosa and their fundic glands, lamina propria, submucosa, mucosa and gastric pits (Fig. 5). Rodents administered **2**–**8** treatment groups displayed variable results, as the gastric epithelium and damage were less in some groups in comparison to the other groups, as displayed in Figs. 6, 7 and 8. Moreover, the immunohistochemical results of antiapoptotic (BCL2) and tumor suppressor (P53) proteins were indicated as brown coloration of the specific immune-stains used. The staining intensity reflected the histologic situation of the damaged tissues; for instance, the staining was very deep or strongly positive for both proteins in the 10% Tween 80-treated group. This result was opposite to the esomeprazole-treated group, which stained mildly or weakly. Likewise, the immune staining of both proteins showed variable intensities in the Compound **2**–**8**-treated groups, as displayed in Figs. 9, 10, 11 and 12. The mechanism underlying apoptosis alteration in gastric damage is complicated since various genes and proteins, including P53 and BCL-2, are involved. BCL-2 expression increases at the early stage of gastric injury and declines during the development of gastric cancer. However, the P53 gene has anticancer roles and triggers apoptosis. Therefore, P53 and bcl-2 expression by cancerous cells could be helpful indicators of gastric cancer^29^.

### Molecular docking study

Preventing gastric acid secretion is the main factor in the inhibition of ulcer formation by antiulcer drugs, such as esomeprazole, a well-known inhibitor of gastric H+/K+-ATPase^30^. Esomeprazole was selected as a valid positive control for the docking results. The interaction affinities of synthesized compounds toward the human gastric H+/K+-ATPase α chain (Swiss-Prot ID: P20648), rat H+/K+-ATPase α protein (Swiss-Prot ID: P09626), and Na+/K+-ATPase crystal structure (PDB ID:2ZXE) have been determined (Table 2).

**Table 2:** Average docking scores (kcal/mol) for plant extract compounds docked into Human H+/K+-ATPase α protein (ID: P20648), Rat H+/K+-ATPase α protein (ID: P09626), and the Na+/K+-ATPase crystal structure (PDB ID:2ZXE).

Compound Name	Human H+/K+-ATPase α protein (ID: P20648)	Predicted Inhibition Constant p*K*i (µM)	Rat H+/K+-ATPase α protein (ID: P09626)	Predicted Inhibition Constant p*K*i (µM)	Na+/K+-ATPase crystal structure (PDB ID:2ZXE)	Predicted Inhibition Constant p*K*i (µM)
	Binding Energy (ΔG) (kcal/mol)		Binding Energy (ΔG) (kcal/mol)		Binding Energy (ΔG) (kcal/mol)	
Esomeprazole	− 7.7	6.1	− 6.8	5.7	− 7.1	5.9
C2	− 6.7	5.7	− 7.0	5.8	− 6.4	5.6
C3	− 7.2	5.9	− 7.7	6.1	− 7.8	6.2
C4	− 6.6	5.6	− 6.8	5.7	− 6.9	5.8
C5	− 6.6	5.6	− 6.2	5.5	− 6.6	5.6
C6	− 6.7	5.7	− 6.9	5.8	− 6.9	5.8
C7	− 10.7	7.4	− 9.0	6.7	− 10.4	7.3
C8	− 8.7	6.6	− 8.5	5.5	− 8.0	6.2

**Figure 13:**
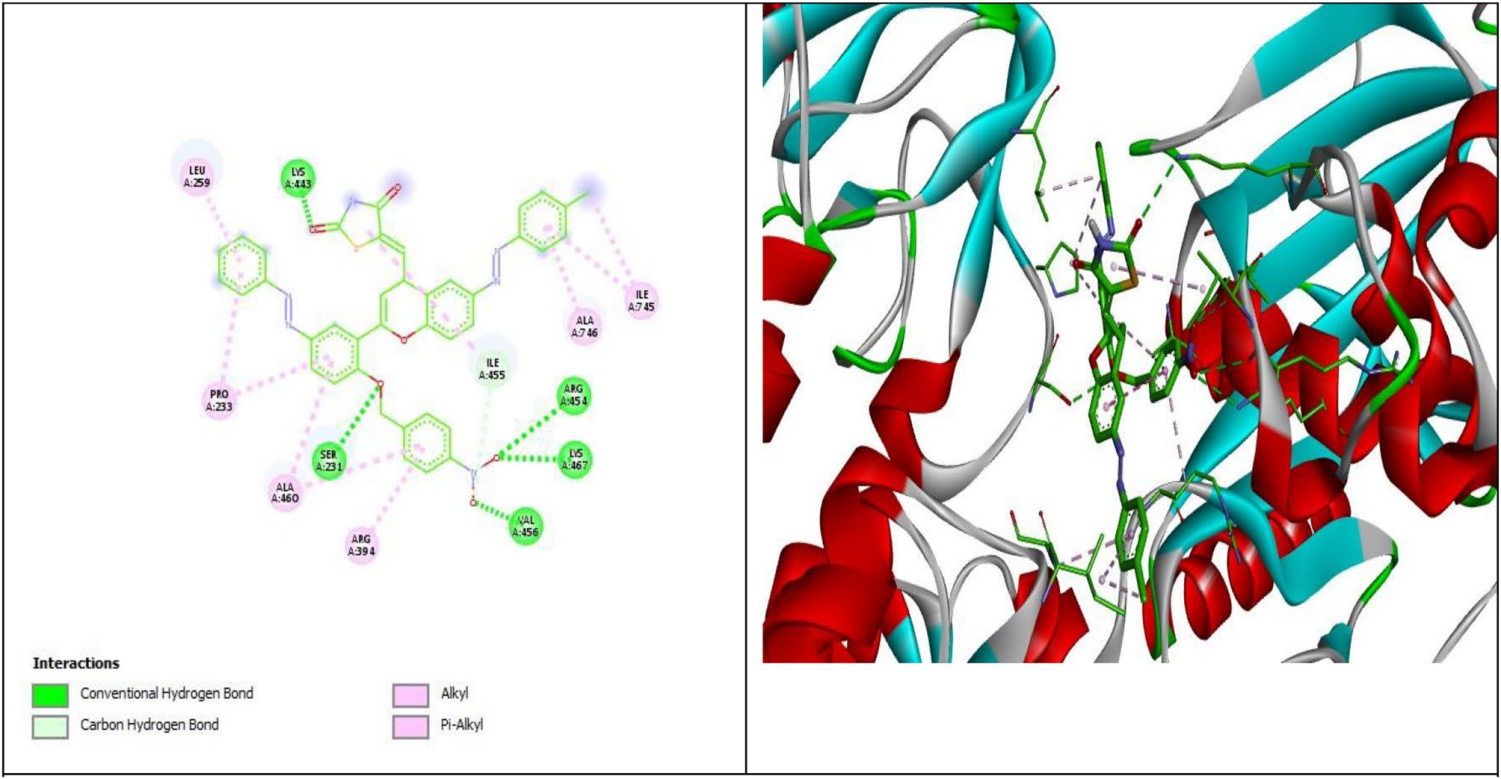
2D and 3D representations of the interaction of **7** with Rat H+/K+-ATPase α protein (ID: P09626).

The lowest free energies and highest affinities were observed for Compounds **7**, **8**, and **3** interactions with the human H+/K+-ATPase α protein (ID: P20648), with docking scores of − 10.7, − 8.7, and − 7.2 kcal/mol, respectively; in contrast, the controlled drug esomeprazole had a docking score of − 7.2 kcal/mol. Similarly, Compounds **7**, **8**, and **3** have the highest docking score interactions with the rat H+/K+-ATPase α protein (ID: P09626) and Na+/K+-ATPase crystal structure (PDB ID:2ZXE). The results showed a degree of similarity in the energy scores between esomeprazole and Compounds **3** and** 4**, while Compounds **7** and **8** exhibited greater affinity toward the H+/K+-ATPase α protein than esomeprazole. The lowest binding sites determined the interaction for Compounds **2**, **4**, **5**, and **6** with the ATPase α protein, with docking scores ranging from − 6.4 to − 6.9 kcal/mol. The binding mode orientation of Compound **7** with the highest binding score shows the formation of an extensive network of hydrogen bonds between Compound **7** and Rat H+/K+-ATPase α protein (ID: P09626) by SER A: 231, ARG A: 454, VALA: 456, ILE A:455 and LYS A: 467. Hydrophobic contact residues were established between **7** and PRO A: 233, ARG A: 394, ALA A: 460, 746, LEU A: 259, and ILE A:745 (Fig. 13).

**Figure 14:**
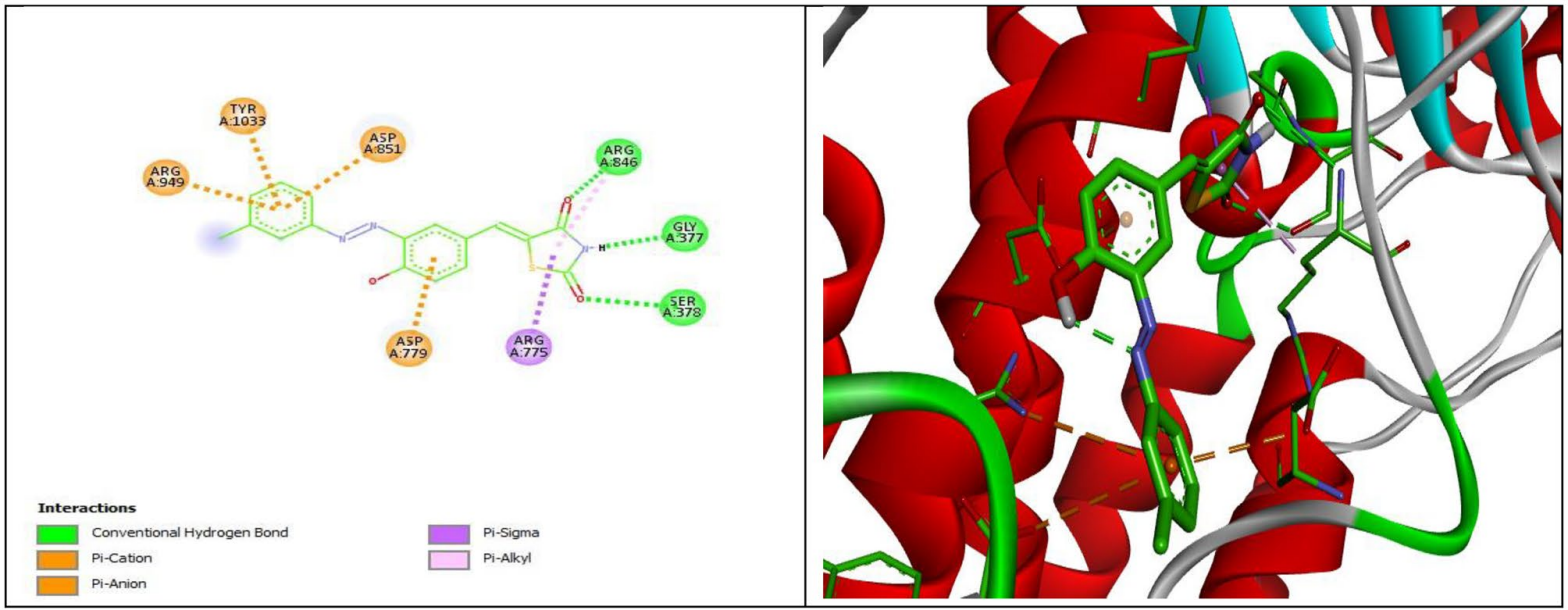
2D and 3D representations of the interaction of **8** with Rat H+/K+-ATPase α protein (ID: P09626).

Compound **8** showed hydrogen bonds with amino acids of ARG A: 846, GLY A: 377, and SER A: 378 of rat H+/K+-ATPase α protein (ID: P09626), with a binding affinity higher than that of the control drug esomeprazole (Fig. 14). Favorable binding site energies were found for **2**, **6**, **4**, and **5,** ranging from − 7.0 to − 6.2 kcal/mol. Esomeprazole interacts through hydrogen bonds with the amino acids GLU A:298, GLN A: 104, and GLN A: 159 present in the rat H+/K+-ATPase α protein (ID: P09626). The other amino acid residues present in the binding pocket of Rat H+/K+-ATPase α protein (ID: P09626) to bond hydrophobic interactions are LEU A: 346, GLU A: 371, ALA A;294, and LYS A: 290.

One of the most common gastric intestinal ailments is gastric ulcers, which affect the human population worldwide^30^.

The highest binding affinity and lowest energy among synthesized compounds were observed for the interaction of **7**, **8**, and **3** with human, rat H+/K+-ATPase α and Na+/K+-ATPase compared with the binding site affinity of the controlled standard drug esomeprazole. Human and rat H+/K+-ATPase α have the lowest binding energy and highest docking score, at **7,** followed by **8** and **3**. On the other hand, favorable docking scores were obtained for Compounds **2**, **4**, **5**, and **6**.

The azo substituents attached at position C-5 of the rhodamine and 2,4-thiazolidinediones in Compounds **3**, **7**, and **8** show high binding site scores and high affinities toward H+/K+-ATPase α and Na+/K+-ATPase compared with Compounds **2**, **4**, **5**, and **6**. Two carbonyl functional Group C=O were present on 2,4-thiazolidinedione derivatives compared to one carbonyl group on rhodamine. Both carbonyl groups show more favorable interactions by hydrogen bonds with residues LYS, ARG, and SER.

#### AMDET analysis

The ADMET study is among the most essential parts of computational drug design. ADMET was estimated for all synthesized compounds using an online web server, i.e., SwissADME (http://www.swissadme.ch/), (https://biosig.lab.uq.edu.au/pkcsm/prediction) and admetSAR (http://lmmd.ecust.edu.cn/admetsar2). The obtained ADMET properties are presented in detail in Table 3.

**Table 3:** List of ADME and physicochemical properties of 5B2T.

#	MW (g/mol)	Caco2+	HIA+	Logp	TPSA A2	nON	nOHNH	RBs	Skin Permeability
	180–500	> 8 × 10–6 cm/s high	< 25 poor > 80 high	< 5	≤ 140	2.0–20.0	0.0–6.0	≤ 10	> **− **2.5 Low skin permeability
2	221.237	0.237	92.084	1.716	89.986	4	2	1	**− **2.777
3	342.40	0.899	90.118	3.691	140.907	5	2	3	**− **3.078
4	266.30	0.789	90.645	2.083	106.042	3	1	2	**− **2.727
5	264.375	1.654	93.277	2.241	109.879	1	1	2	**− **2.397
6	266.30	0.786	90.827	2.083	106.042	3	1	2	**− **2.733
7	808.06	**− **0.583	100	11.804	319.129	10	1	10	**− **2.735
8	339.37	0.662	88.791	4.439	141.855	5	2	3	**− **3.088

MW, molecular weight; BBB+, blood–brain barrier; Caco2+, Caco-2, permeability; HIA+, %human intestinal absorption; logp, logarithm of partition coefficient between n-octanol and water; TPSA2, topological polar surface area; nON, number of hydrogen bond acceptors; nOHNH, number of hydrogen bond donors; RBs, number of rotatable bond.

All synthesized compounds exhibit acceptable ADMET molecular weight properties in the range of 221.237–342.40 (g/mol) (< 500), except Compound **7** with a molecular weight of 808.06 (g/mol), which was observed as a violation in proportion to Lipinski, Ghose, and Muegge rules. A significant value of 78% revealed a high chance of crossing the blood brain barrier. The percentage of human intestinal drug absorption was found to be in a significant range from 88.791 to 100, which was in the acceptable range (> 80). Octanol–water partition coefficients (Log P) were found to be less than 5 for all compounds, with one violation for Compound 7 based on the Lipinski rule MLOGP > 4.15. The topological surface areas (TPSA) were in the acceptable range for Compounds 2, 4, 5, and 6 (< 140) and Compound 3, and 8 passed over an acceptable range of 140 A^0^. A significant TPSA 319.129 A^0^ was found for Compound **7,** which was considered a violation according to Veber, Egan, and Muegge rules. In addition, H-bond acceptors (nON) and donors (nOHNH) were found to be in the range of 1–10 and 1–2, respectively (Table 3).

## Conclusions

Rhodanine and 2,4-thiazolidinedione scaffolds clearly established curative properties in the healing of ulcers induced by ethanol. Inhibition percentage and ulcer areas are displayed for synthesized compounds. P53 and BCL-2 expression could be supportive markers of healing of gastric lesions. Molecular docking results of active constituents of synthesized compounds revealed favorable binding affinity against H+/K+-ATPase α protein and Na+/K+-ATPase with the highest binding score of HO^−^ substituent of 2,4-Thiazolidinediones and higher molecular structure of Rhodanine Scaffolds.

### Supplementary Information

Supplementary Figures.

## Data Availability

The authors declare that the data supporting the findings of this study are available within the paper and its Supplementary Information files. Should any raw data files be needed in another format they are available from the corresponding author upon reasonable request. Source Data are provided with this paper.
