# Supplementary material for: Gastroprotective effect of rhodanine and 2,4-thiazolidinediones scaffolds in rat stomachs by contribution of anti-apoptotic (BCL-2) and tumor suppressor (P53) proteins
Source: Sci Rep. 2024 Jan 19;14:1699. doi: 10.1038/s41598-024-51446-4 (PMC10799065; doi:10.1038/s41598-024-51446-4)
Supplement: Supplementary file 1 — Supplementary Figures. [file 41598_2024_51446_MOESM1_ESM.pdf]

supplementary file

Gastroprotective effect of rhodanine and 2,4-thiazolidinediones Scaffolds in rat stomachs by contribution of anti-apoptotic (BCL-2) and tumor suppressor (P53) proteins

Rozh Q. Amin[1] , Zahra A. Amin[2], Hiwa O. Ahmad[3, 5*], Diler D. Ghafur[4] , Melodya G. Toma[3], Nyan Sabah[2], Muhammad Fakhir[2], Gardoon Abdulla[3]

[1] Department of Pharmacy, Paitaxt Technical Institute, Erbil, Kurdistan region, Iraq.

[2] Department of Clinical Analysis, College of Pharmacy, Hawler Medical University, Erbil, 44001, Kurdistan region, Iraq.

[3] Department of Pharmaceutical Chemistry, College of Pharmacy, Hawler Medical University, Erbil, 44001, Kurdistan region, Iraq.

[4] Department of Chemistry, College of Education, Salahaddin University, Erbil, Kurdistan region, Iraq.

[5] Pharmacy Department, College of Medicine, University of Kurdistan- Hewlêr, Kurdistan region, Iraq.

(*) Corresponding author: hiwa.omar@hmu.edu.krd

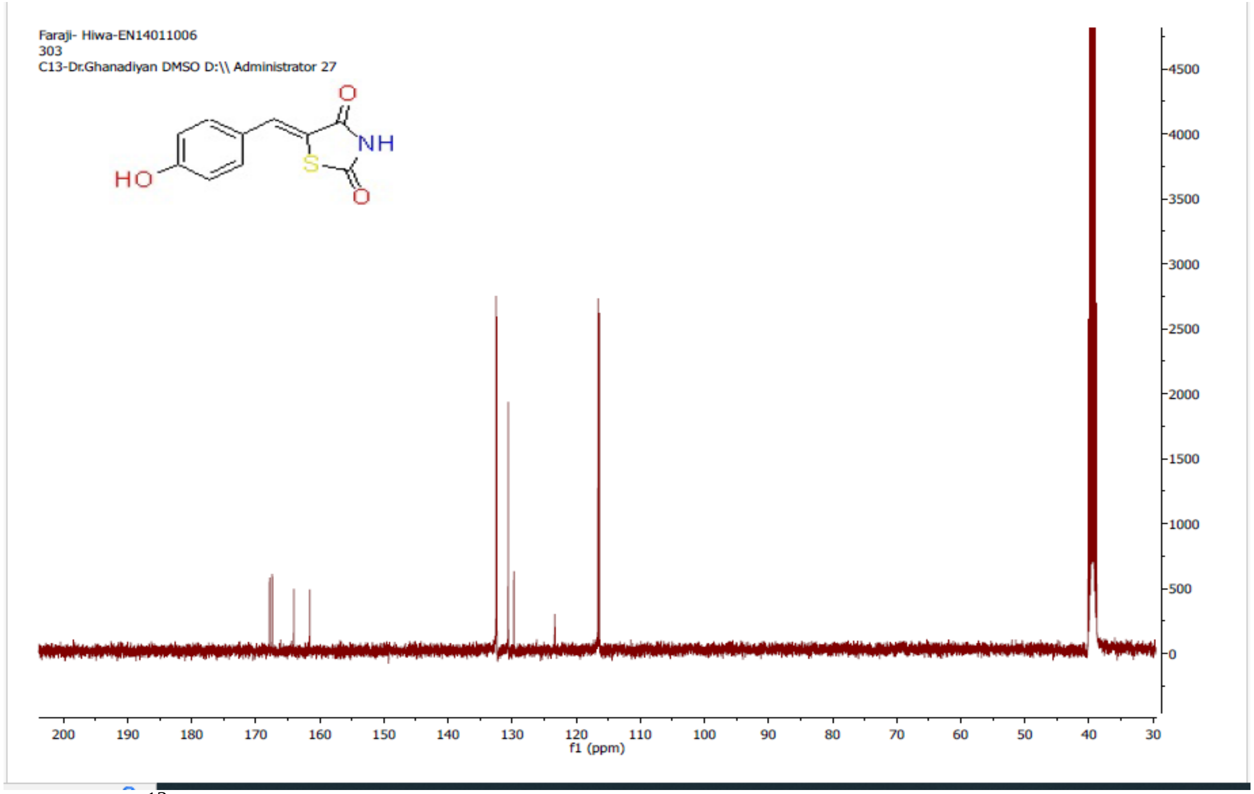

Figure S2: ^{13}C-NMR spectra of compound **2**

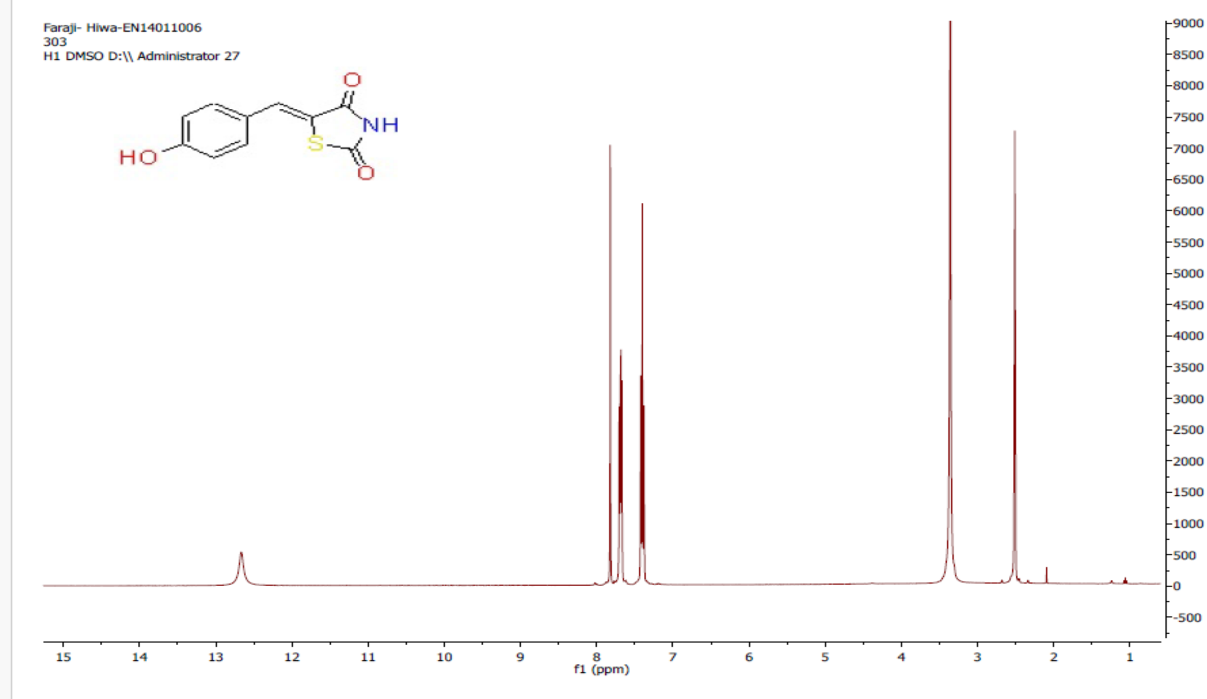

Figure S1: ^{1}H-NMR spectra of compound **2**

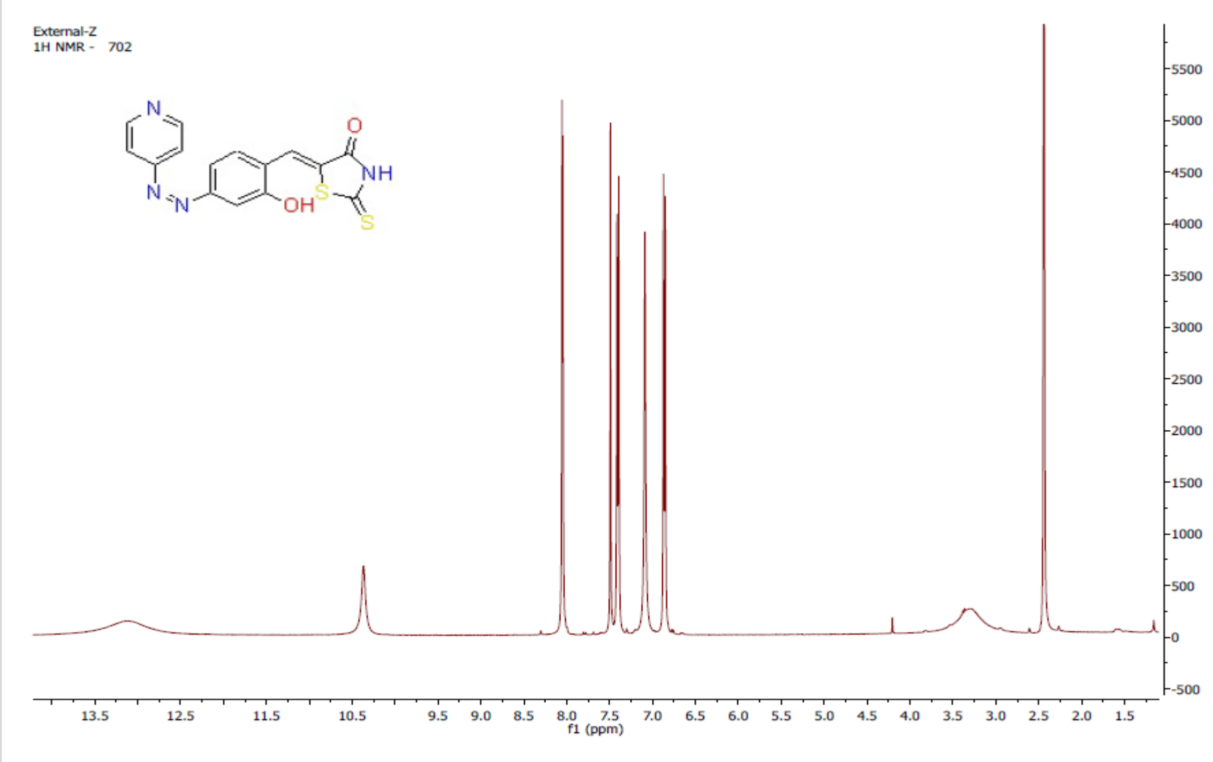

Figure S3: ^{1}H-NMR spectra of compound **3**

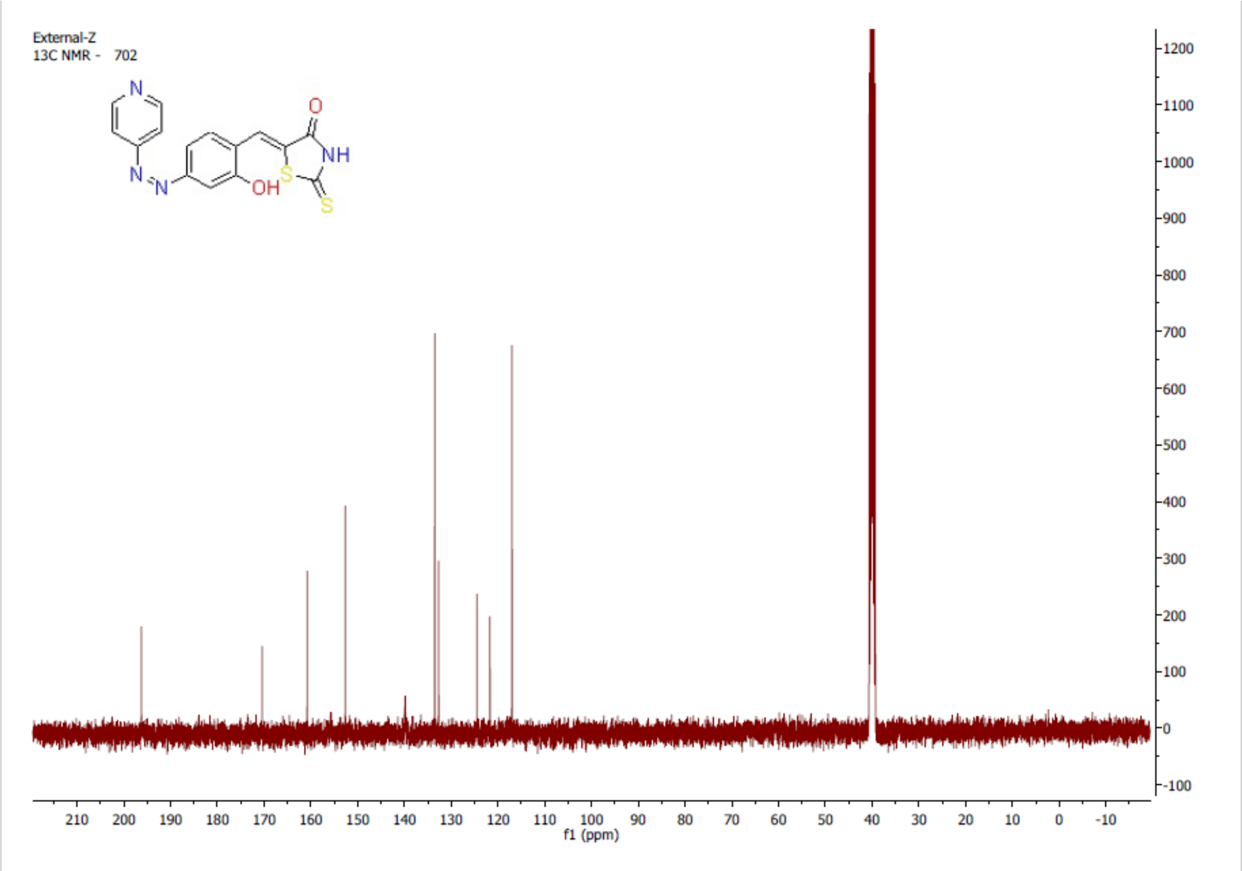

Figure S4: ^{13}C-NMR spectra of compound **3**

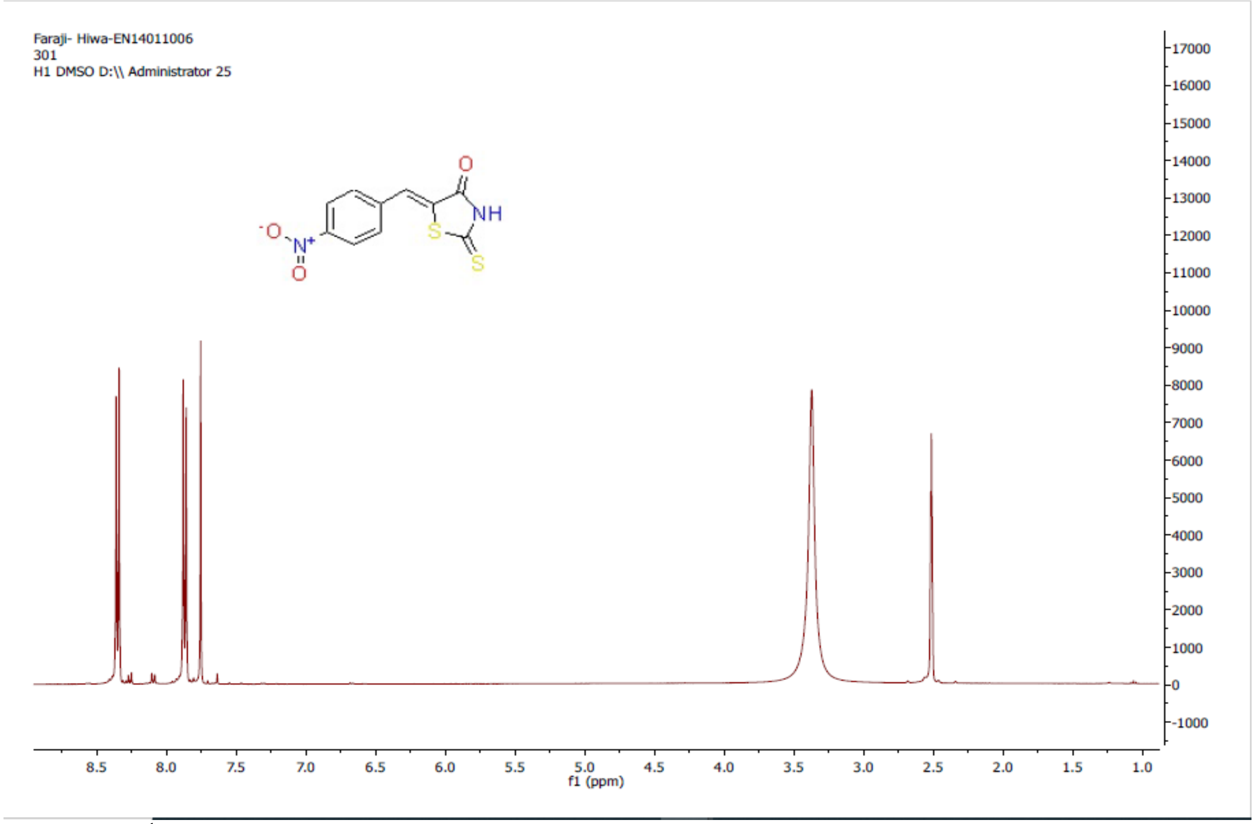

Figure S5: ^{1}H-NMR spectra of compound **4**

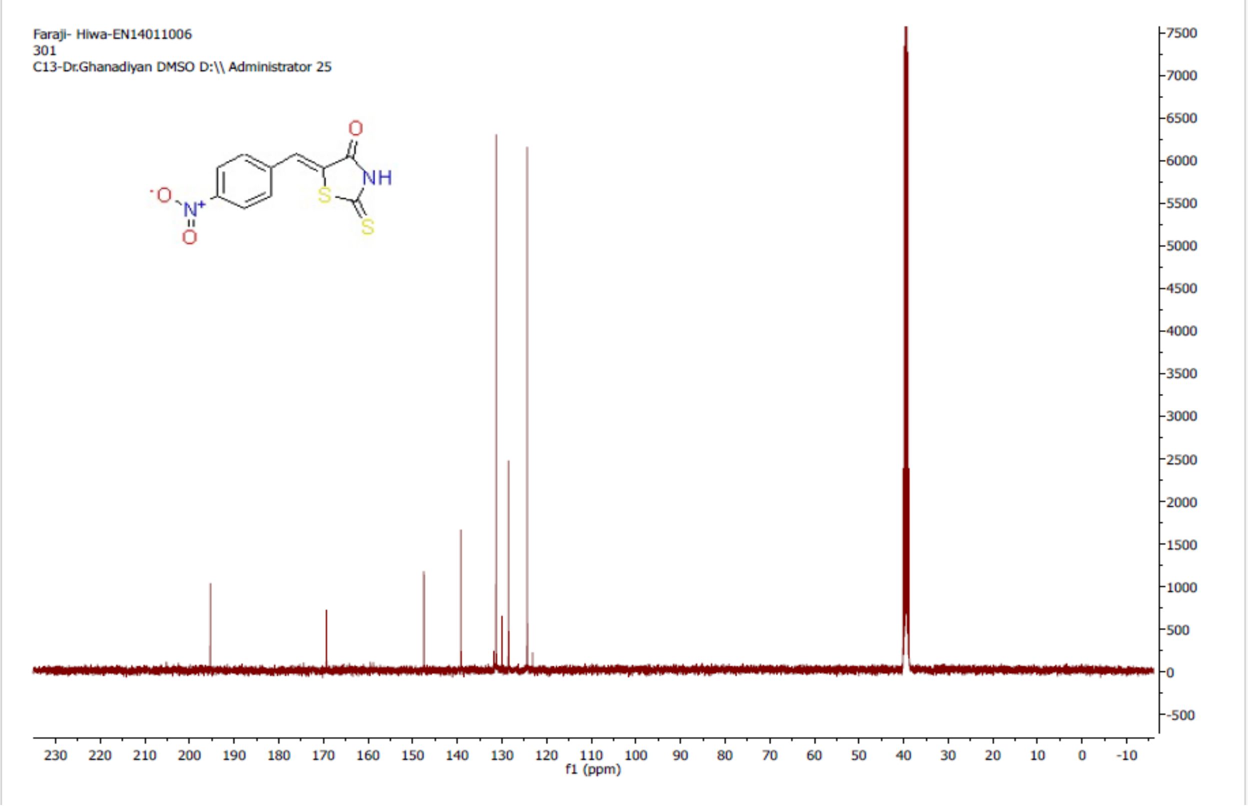

Figure S6: ^{13}C-NMR spectra of compound **4**

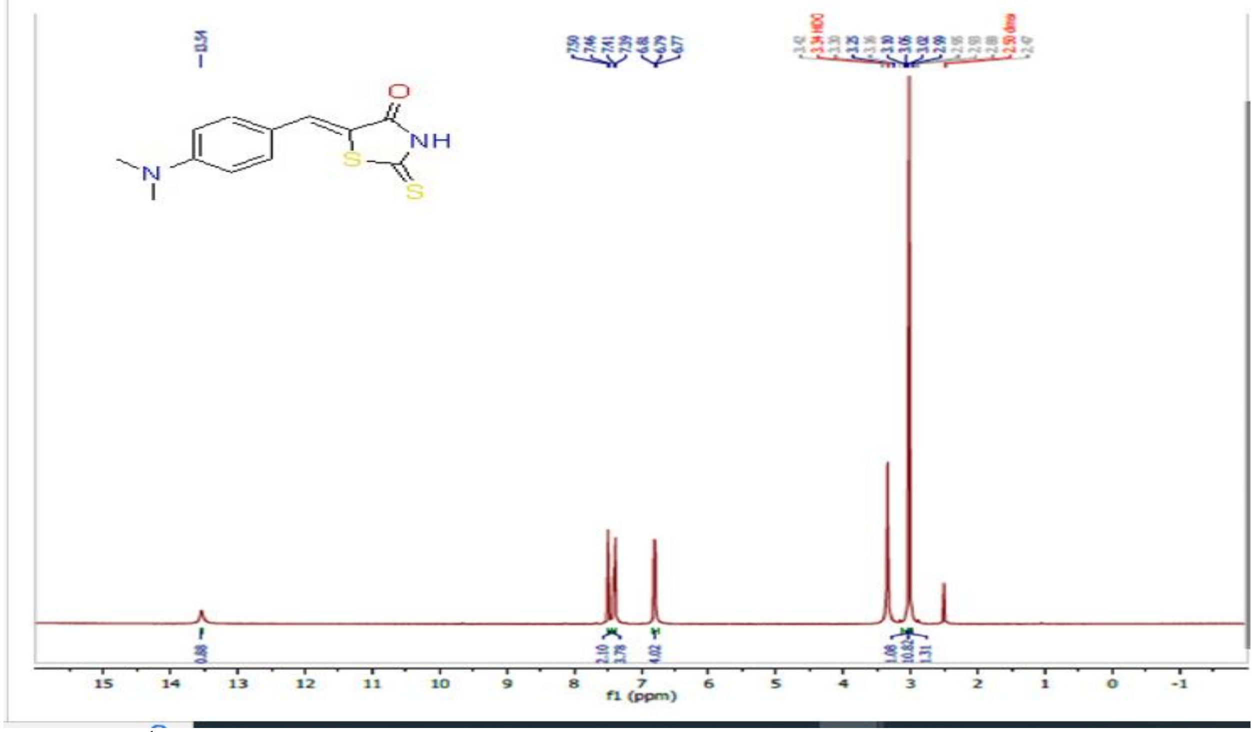

Figure S7: ^{1}H-NMR spectra of compound **5**

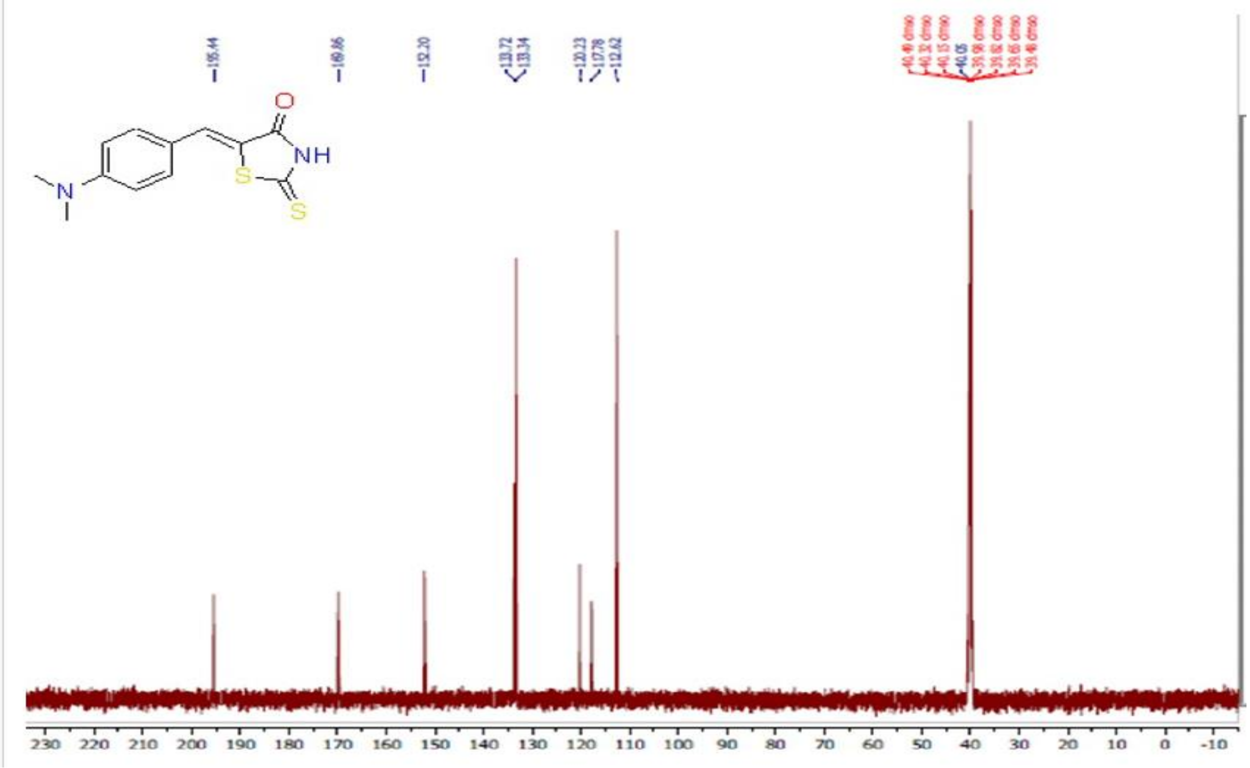

Figure S8: ^{13}C-NMR spectra of compound **5**

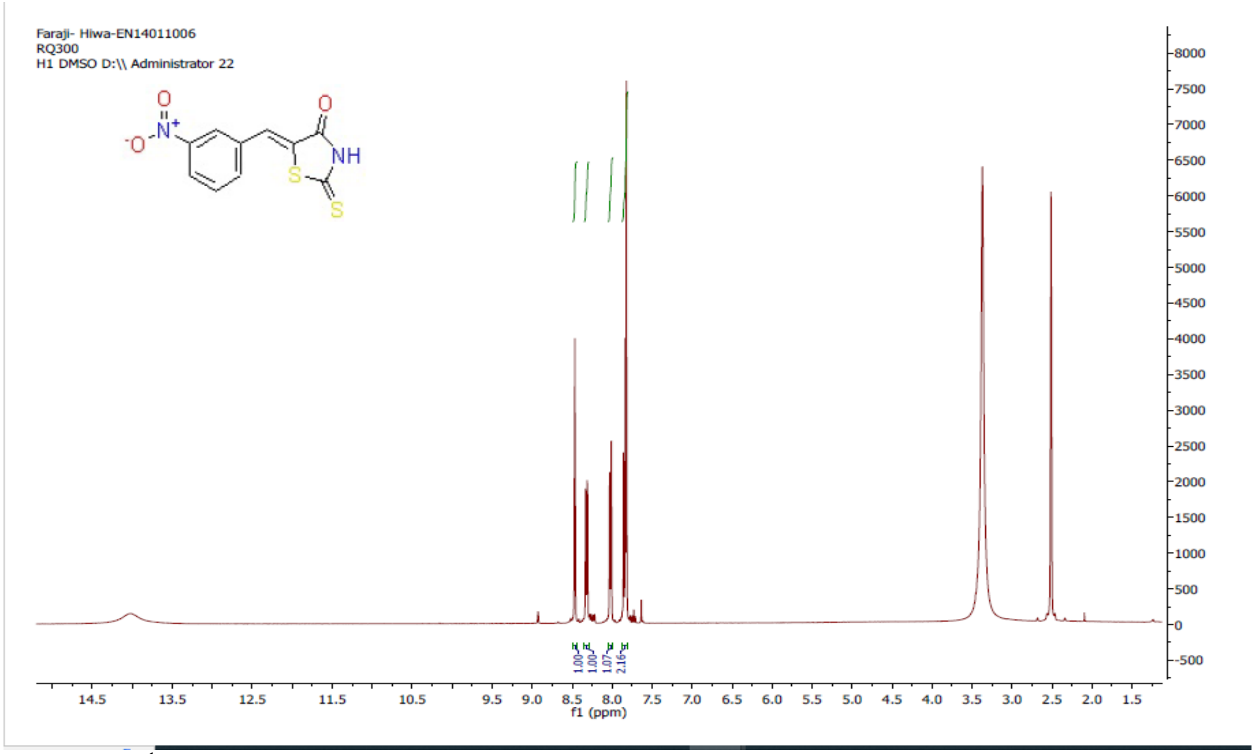

Figure S9: ^{1}H-NMR spectra of compound **6**

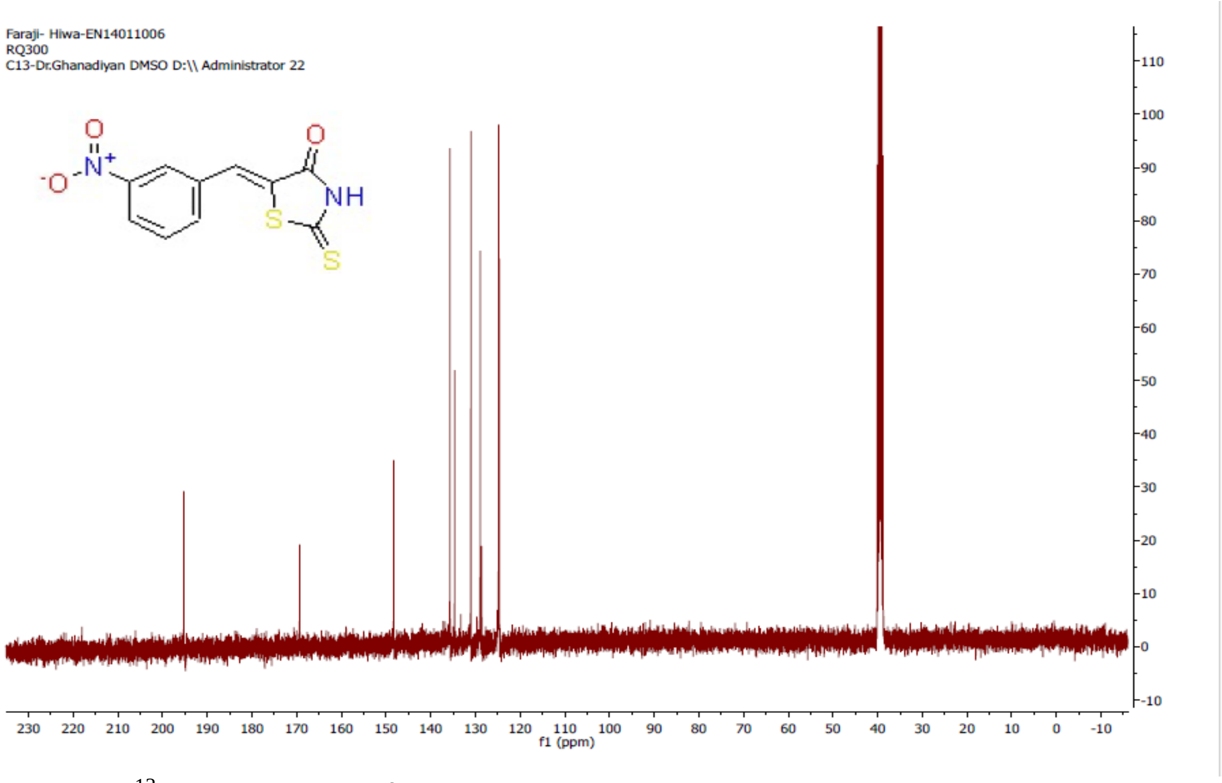

Figure S10: ^{13}C-NMR spectra of compound **6**

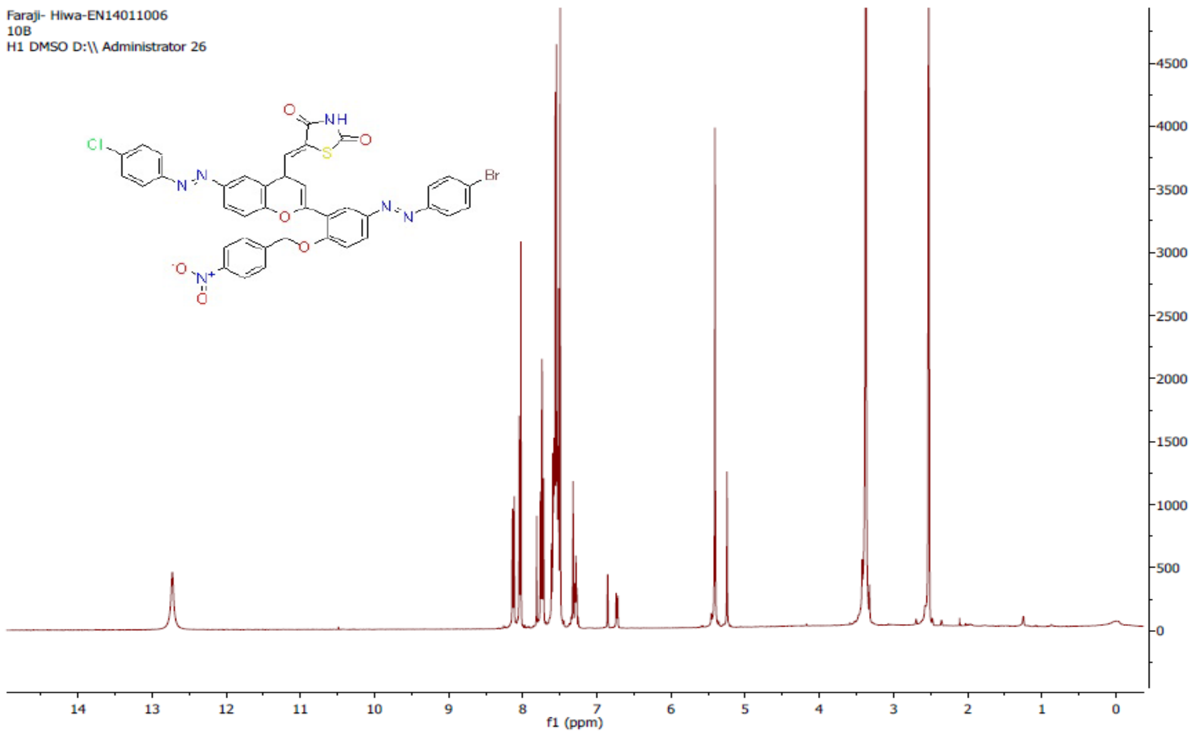

Figure S11: ^{1}H-NMR spectra of compound **7**

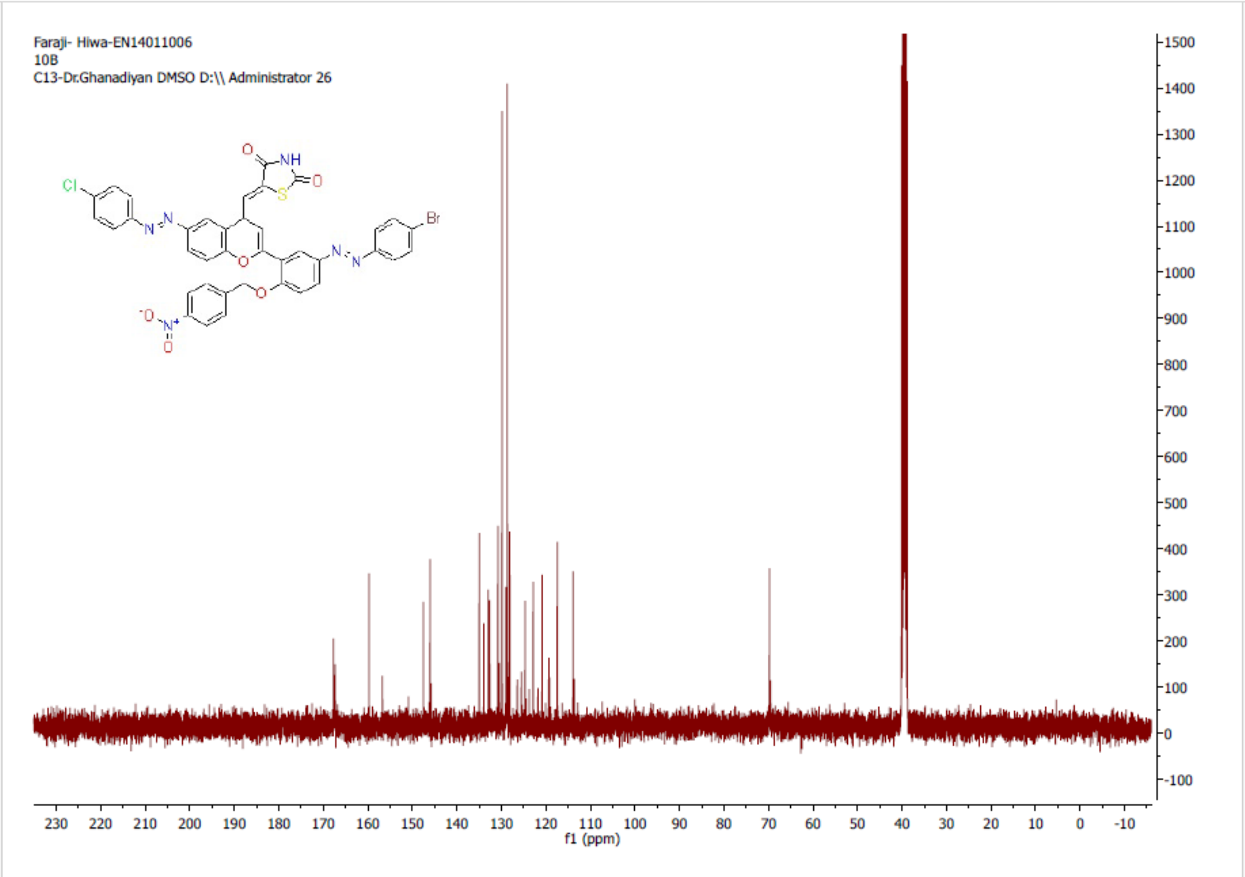

Figure S12: ^{13}C-NMR spectra of compound **7**

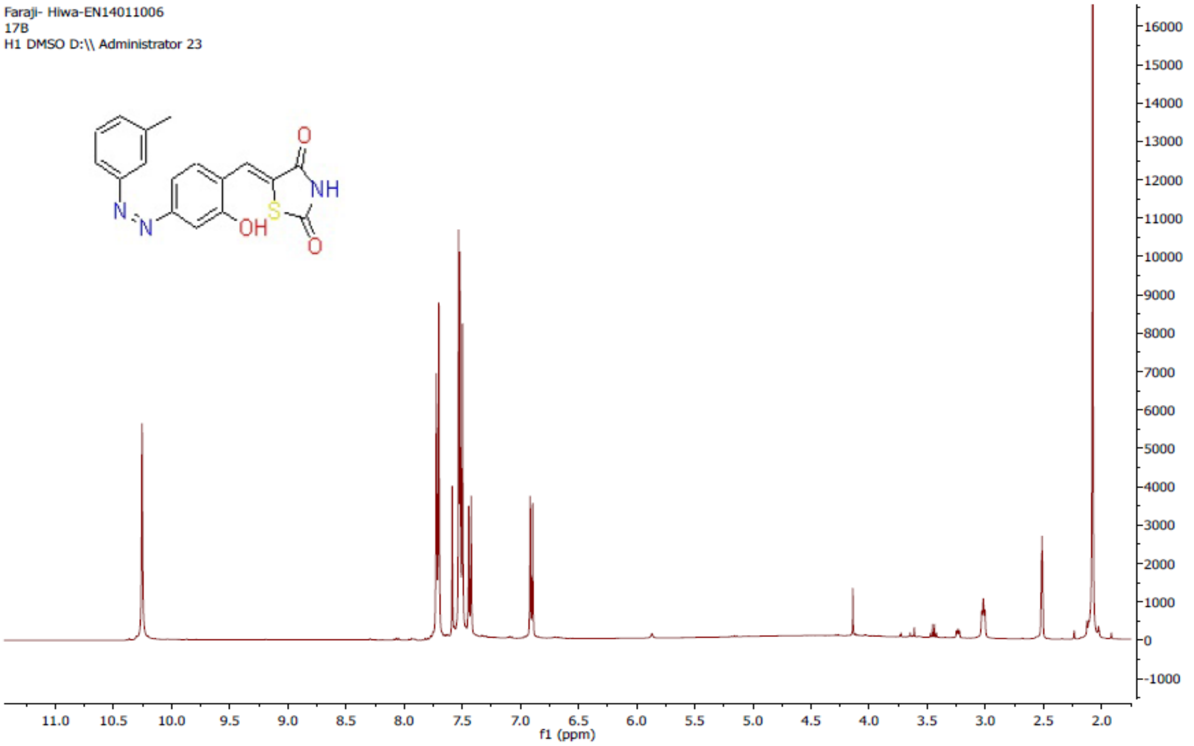

Figure S13: ^{1}H-NMR spectra of compound **8**

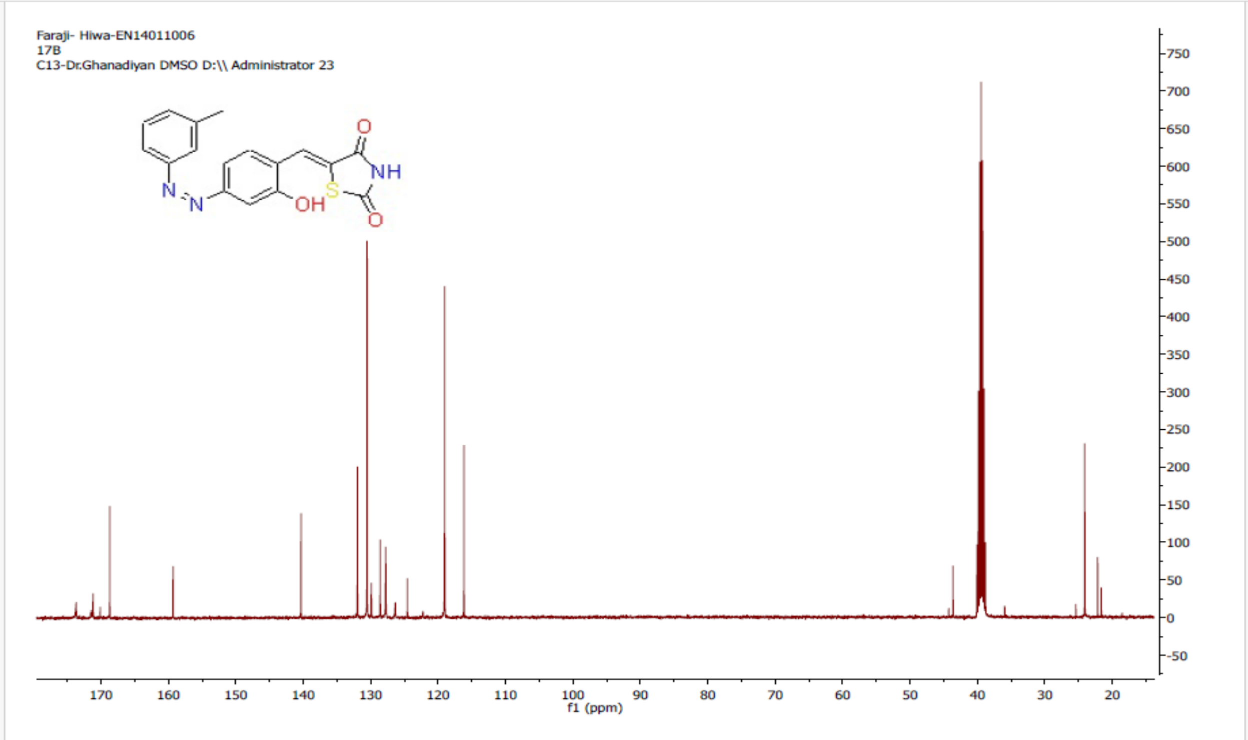

Figure S14: ^{13}C-NMR spectra of compound **8**
